# Increasing the Depth of Current Understanding: Sensitivity Testing of Deep-Sea Larval Dispersal Models for Ecologists

**DOI:** 10.1371/journal.pone.0161220

**Published:** 2016-08-30

**Authors:** Rebecca E. Ross, W. Alex M. Nimmo-Smith, Kerry L. Howell

**Affiliations:** School of Marine Science and Engineering, Plymouth University, Plymouth, United Kingdom; Bangor University, UNITED KINGDOM

## Abstract

Larval dispersal is an important ecological process of great interest to conservation and the establishment of marine protected areas. Increasing numbers of studies are turning to biophysical models to simulate dispersal patterns, including in the deep-sea, but for many ecologists unassisted by a physical oceanographer, a model can present as a black box. Sensitivity testing offers a means to test the models’ abilities and limitations and is a starting point for all modelling efforts. The aim of this study is to illustrate a sensitivity testing process for the unassisted ecologist, through a deep-sea case study example, and demonstrate how sensitivity testing can be used to determine optimal model settings, assess model adequacy, and inform ecological interpretation of model outputs. Five input parameters are tested (timestep of particle simulator (TS), horizontal (HS) and vertical separation (VS) of release points, release frequency (RF), and temporal range (TR) of simulations) using a commonly employed pairing of models. The procedures used are relevant to all marine larval dispersal models. It is shown how the results of these tests can inform the future set up and interpretation of ecological studies in this area. For example, an optimal arrangement of release locations spanning a release area could be deduced; the increased depth range spanned in deep-sea studies may necessitate the stratification of dispersal simulations with different numbers of release locations at different depths; no fewer than 52 releases per year should be used unless biologically informed; three years of simulations chosen based on climatic extremes may provide results with 90% similarity to five years of simulation; and this model setup is not appropriate for simulating rare dispersal events. A step-by-step process, summarising advice on the sensitivity testing procedure, is provided to inform all future unassisted ecologists looking to run a larval dispersal simulation.

## Introduction

Dispersal has many important ecological functions in regulating the structure of a population. These functions have consequences for species survival and evolution [1]. Dispersal therefore has an impact upon conservation and management decisions and is a pivotal factor in the establishment of self-sustaining marine protected area networks worldwide.

Biophysical modelling is a well-established technique in shallow water dispersal studies. In this context biophysical modelling is defined as the use of hydrodynamic data (e.g. in situ ADCP data or the outputs of hydrodynamic models) combined with biological parameters to advect theoretical particles representing animals in order to predict dispersal patterns. This technique has been applied to studies of e.g. jellyfish [2], juvenile turtles [3], larval fish [4], and larval invertebrates [5] in order to discern the influence of ocean currents on faunal dispersal abilities. A number of review articles are available in this field to familiarise any ecologist with suitable methods and their requirements (e.g. [6–9], with [8] specifically addressing the bio- components such as mortality and larval behaviour—factors which are not addressed in this paper).

In the deep-sea however biophysical modelling is still in its infancy due to the paucity of well resolved biological (e.g. larval behavioural data, mortality estimates, swimming speeds, buoyancy, etc.) and oceanographic data required to drive dispersal simulations. To date, deep-sea studies are mostly focussed on vent and seep fauna (e.g. [10–13]), with a few more recent studies beginning to apply biophysical models in other settings (e.g. polyplacophoran wood-fall specialists [14], sedimented slope echinoids [13], protobranch bivalves [15], source-sink hypothesis [16]). Most of these studies required the assistance of a physical oceanographer to build and run the models.

Now, with the availability of reasonably resolved hydrodynamic model outputs and custom particle tracking software designed specifically to simulate larval dispersal, the number of deep-sea studies is likely to increase. Fossette et al. [17] provide an overview of potential sources of hydrodynamic data, and the supplementary data associated with Hilario et al. [18] reviews some of the offline particle tracking software suited to larval dispersal simulations. These tools could be used without the additional assistance of a physical oceanographer, but there is the risk that ecologists may be faced with a black box: a model which appears to work but whose inner workings are unknown, potentially resulting in misuse and misunderstanding of the models capabilities. This paper hopes to offer some guidance to those ecologists who, by design or necessity, choose to “fly solo”.

While user manuals and literature written on specific model builds may elucidate many of the model’s inner workings, sensitivity testing–the permutation of model input parameters to observe the result in model outputs–can provide practical insight into the workings of the model, define limits on input parameter values, and temper expectations of what conclusions can be drawn from simulations [19]. Furthermore existing publications provide some insight into shallow water sensitivity tests [20] and cautionary tales regarding model temporal and spatial resolution [21] but to date there is little advice catering to deep-sea model users who may encounter additional challenges.

There are three critical caveats of biophysical modelling that need to be understood before undertaking a modelling study: 1) by definition a model is a simplification of reality and therefore cannot be expected to represent every process adequately, 2) hydrodynamic models are usually built by and for physical oceanographers and therefore are not tailored to the needs of larval dispersal modelling and will require some compromise on the part of the ecologist, 3) there is usually a trade-off between model quality and computational power (and this applies to both the hydrodynamic model and the particle simulator). These issues are compounded when working in the deep-sea. The potential for longer planktonic larval durations due to metabolic constraints in cold deep sea waters [22–24] requires that models span larger areas, over greater depth ranges than shallow water/coastal studies. Furthermore these locations are usually offshore and therefore lacking in high resolution data. Hydrodynamic models which best fulfil this requirement are currently based on topographic maps derived from altimetry readings: a method with poorest topographic accuracy over areas of deep water and thick sediment seafloor. New topographic maps (not yet used in existing hydrodynamic models) were produced in 2014 improving existing maps by two to four times resolution, yet still these are only able to detect seamounts 1-2km tall [25]. As topography induces many hydrodynamic features, if the topography is inaccurate or coarsely resolved the hydrodynamics will also suffer. The need to cover large areas of ocean demands coarsened resolution due to computational restrictions necessitating temporal and spatial averaging. This averaging process further reduces the accuracy of the hydrodynamics [21] especially when considering the scales of relevance to a microscopic larva [8].

The environment at depth is often considered more stable than in surface waters but there are still many turbulent events. Benthic storms, which are caused by turbidity currents and deep penetrating eddies, may occur eight to ten times a year [26] but they are unlikely to be represented within dispersal simulations employing large scale hydrodynamic models. This simplified view of deep water is perpetuated in standard model output structures such as the Levitus convention of data structuring where the deeper you go the coarser the output resolution (in Levitus one data point is output every 50m at 150m-300m depth, every 100m at 300m-1500m, every 250m at 1500m-2000m, and every 500m from 2000m-5500m depth). Therefore biophysical models run from model outputs may result in decreasing sensitivity of parameters with depth due to the ever coarser resolution of output data points, between which the simulator must interpolate.

Beyond the trade-offs already built into the construction of the hydrodynamic model, the running of a particle simulator can place heavy demands on computational effort and analysis time [9]: a problem that would usually be the job of the physical oceanographer to solve, but which would fall to the ecologist if working alone. All of the parameters tested in this study affect the two most computationally intensive aspects of the simulation–the total number of particles being simulated and the number of velocity fields being loaded into the simulator. It should therefore be a high priority to optimise these parameters. The modeller’s aim is to find a balance between obtaining a saturated state within the model, where you have fulfilled the full potential of the models predictive power, whilst not including redundant autocorrelated simulations which are wasteful of computational power and analysis effort.

Knowing these caveats exist (and more besides, see [6–9, 18]), it is important that ecologists explore the capabilities and limitations of their model setup before undertaking an ecological study. The inputs should be tailored to the structure of the model and expectations should be tempered as to what model outputs may realistically represent. Complementing the work of Simons et al [20], this study explores the sensitivity of several parameters, all of which may be affected more severely than in shallow-water studies. Note that additional parameters are covered by Simons et al [20], and neither list is exhaustive of what could or should be tested. While other literature touches on the sensitivity testing of model parameters (e.g. [27–30]), the purpose of this study is to provide a more step-by-step approach for those ecologists faced with setting up their first larval dispersal model:

The aims of this study are:

1. To describe methods suited to detecting spatial autocorrelation due to model structure and assessing model saturation (which is similar to undertaking a power analysis)
2. To show how these methods can be used to optimise model inputs and assess model adequacy
3. To highlight the ecological consequences of parameter settings and identify deep-sea specific issues to benefit all future larval dispersal research
4. To provide a clear step-by-step procedure for other ecologists to follow when setting up their first larval dispersal model

## Methods

### Study Area

**Fig 1:**
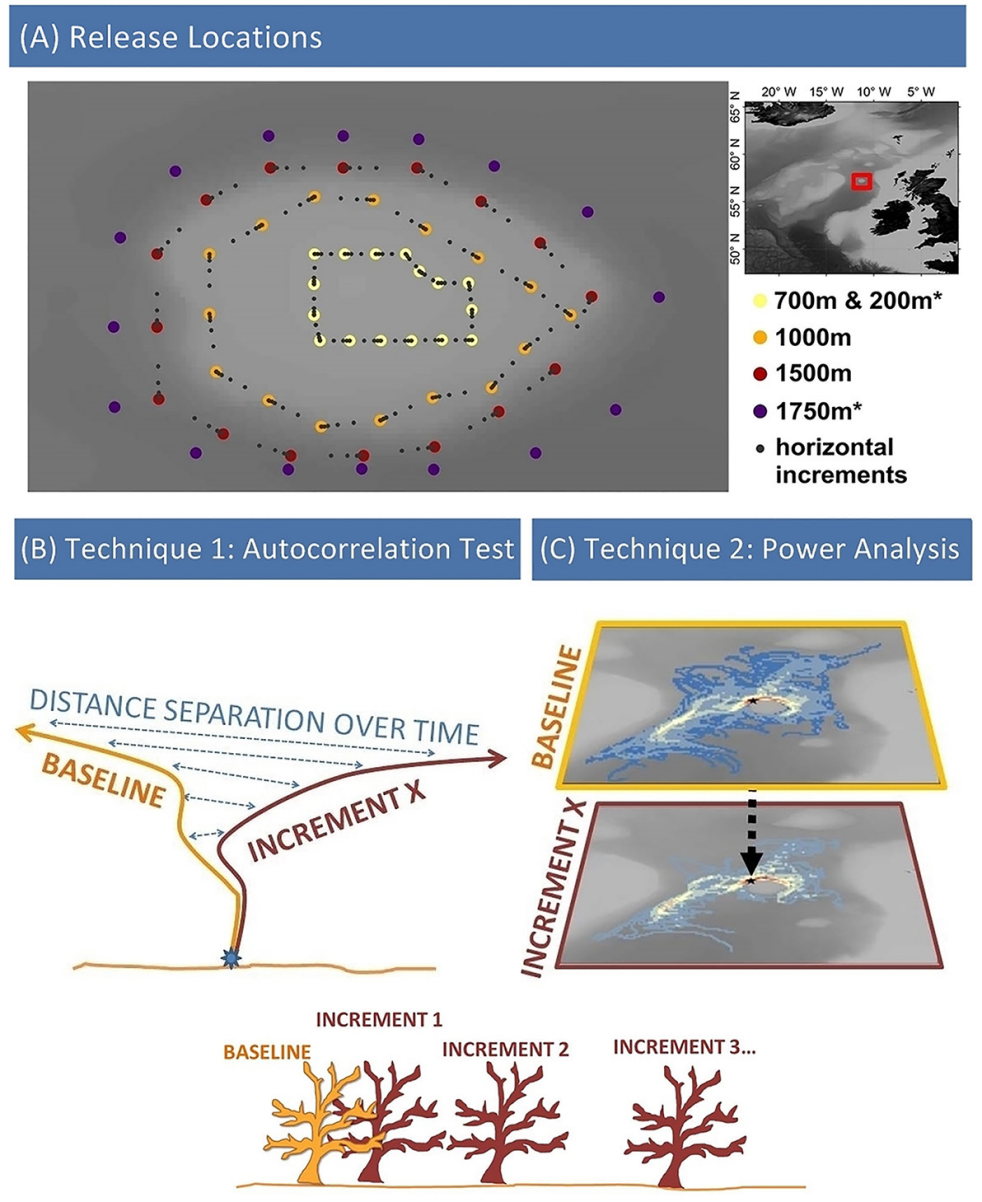
Methods used in this study. The study area (A) focused on Anton Dohrn Seamount (ADS) in the Rockall Trough region East of UK and Ireland. Release locations were defined based on model topography and equally spaced around the circumference of ADS at three standard depths (700m, 1000m, 1500m) with modified depths for the vertical separation test (200m and 1750m; marked with asterisk *) and increment locations shown for the horizontal separation test (coordinates given in S1 File). Two analysis techniques were used in this study: (B) The Autocorrelation tests are a comparison of each increment track with its corresponding baseline in terms of distance separation over time. (C) Power Analysis tests derive a linear correlation between rasters of track density, converting this into the fraction of unexplained variance metric (after Simons et al. [20]). Bathymetry and topography data were obtained online from the GEBCO Digital Atlas published by the British Oceanographic Data Centre on behalf of IOC and IHO, 2003 (GEBCO 30 arc-second grid, www.gebco.net).

The Northeast Atlantic, in offshore waters west of the UK and Ireland, is used as a case study. The region (Fig 1), centred on the Rockall Trough (RT), has been a hotbed for deep-sea research for over a century and therefore offers a range of historic datasets which can be used for preliminary groundtruthing. The region’s currents and water mass structures are well documented (e.g. [31, 32]).

Situated in the centre of the RT, Anton Dohrn Seamount (ADS) was selected as the focal point for the study providing a site for amphi-directional releases across a wide depth range in order to best capture the currents in the area. ADS is a guyot (table mount) with a summit at 521m, and a maximal depth in the South at approximately 2100m, although within the coarse bathymetry of the hydrodynamic model it extends between 600m and 2000m depth. ADS is also a focal point in Holliday et al.’s [31] observational data for the region which spans 23 years of recordings at 22 full-depth standard location stations.

### Hydrodynamic model

Freely available outputs from HYCOM+NCODA GLBa0.08 numerical model were used to provide the velocity fields which drive the particle simulator (hycom.org, [33]). Daily averaged data from 2008–2013 were used in the TR tests, while all other tests are based on the data from 2012–2013. HYCOM lends itself well to deep-sea studies due to its unique hybrid vertical grid structure within the native model (data is aligned with isopycnals in the open ocean, transforming to terrain-compressing (aka “sigma grid”) layers over topography). The accessible outputs however average the native data into a list of depths (aka a “z-level grid”), specifically following the aforementioned Levitus depth structure. This study does not run a comparison between the native (online) and output-based (offline) particle simulation methods, but does explore the capabilities and limitations of this common output structure. The offline output-based method is the most accessible to deep-sea ecologists [17].

Vertical velocity is not output as standard from HYCOM, and is not available in the HYCOM outputs used by this study. It can be calculated separately based on the continuity equation, but this parameter is known to be noisy, problematic, and would be based on an interpolated grid (the output z-level grid) different from the native model structure (a hybrid grid). Therefore vertical velocity was consciously excluded from this study. This is further justified in this case due to average background vertical velocity in the deep ocean being estimated at 10^−5^ cm s^-1^ (i.e. <1m in 100 days) [34], but should this variable be available we would advise its inclusion, especially when conducting simulations in shallower water. Note that test results are likely to be affected by the inclusion of vertical velocity vectors.

The HYCOM Global analysis outputs project their data onto a Mercator horizontal grid for the majority of the world, but north of 47°N they adopt an atypical bi-polar grid. While the study area falls within this potentially problematic region, the particle simulator model used in this study has a facility to re-project the hydrodynamic data into a Mercator grid prior to simulations, and was tuned for use with HYCOM specifically (but can be used with other model outputs); this re-gridding facility was used during this study. Note that the more recently available HYCOM Global reanalysis data is already projected onto a Mercator grid north of 47°N.

### Particle Simulator

The freely available Connectivity Modeling System (CMS) is a recently-developed offline Lagrangian particle simulator (https://github.com/beatrixparis/connectivity-modeling-system, [35]). It was especially developed for larval dispersal modelling, with multiple modules available for the integration of biotic and abiotic data, and is under continual development with additional modules becoming available for specialist uses. CMS has the facility to interface with the HYCOM servers and download hydrodynamic data directly. It can also utilise z-level stored hydrodynamic data whilst providing a re-gridding routine to adapt any data in problematic formats (e.g. uncommon non-orthogonal projections as mentioned above).

CMS and HYCOM together have already been used as the basis of multiple studies within different fields (including non-biological) and have been employed in studies of coral reef connectivity (e.g. [36–38]). This study uses the CMS in its simplest configuration: as a passive particle simulator. It uses a fourth order Runge-Kutta method of advection, and prioritises a tricubic interpolation method through space, although will alter this to tri-linear in the vicinity of land, or bicubic if run on a 2D basis (as is used here). A linear interpolation is run between time snapshots to advect the particle through changing velocity fields.

It should be noted that this study does not test the number of larvae released per spawning event as the model set up used here does not parameterise diffusivity. Without diffusivity all larvae released in one spawning event would follow identical tracks. It is possible to add diffusivity, but in the CMS it would require two arbitrary nest-wide values (one horizontal, one vertical) which themselves require sensitivity testing and careful study-specific consideration. Diffusivity should be tested and used in studies seeking to simulate multiple larvae per spawning event. It is not tested here as other particle simulators may handle this differently.

### Parameters

The following parameters were selected for testing in this study:

- Timestep (TS) of particle simulator
- Horizontal Separation (HS) of release points
- Vertical Separation (VS) of release points
- Release Frequency (RF)
- Temporal Range (TR) of hydrodynamic data

The first three parameter tests (TS, HS and VS) aim to detect spatial autocorrelation as a product of model structure. In dispersal models, every particle run is expected to provide useful data, but particles released too close together may show related or identical outcomes entirely due to their spatial proximity. This is because model data is gridded (the model resolution defines the distance between data points) essentially causing data to act as if it is categorical rather than continuous. This is true for fine or coarse resolutions and for any interpolations applied; there will always come a point where a release location will be positioned within the same effective grid cell/category as another, thereby acting as a duplicate whose outcome is both unnecessary and unmeaningful. The aim of these tests is to ensure that all release positions simulated will represent independent samples from which ecological conclusions can be drawn. Therefore we ask: what is the highest resolution parameter setting that does not result in spatially autocorrelated outputs?

**Fig 2:**
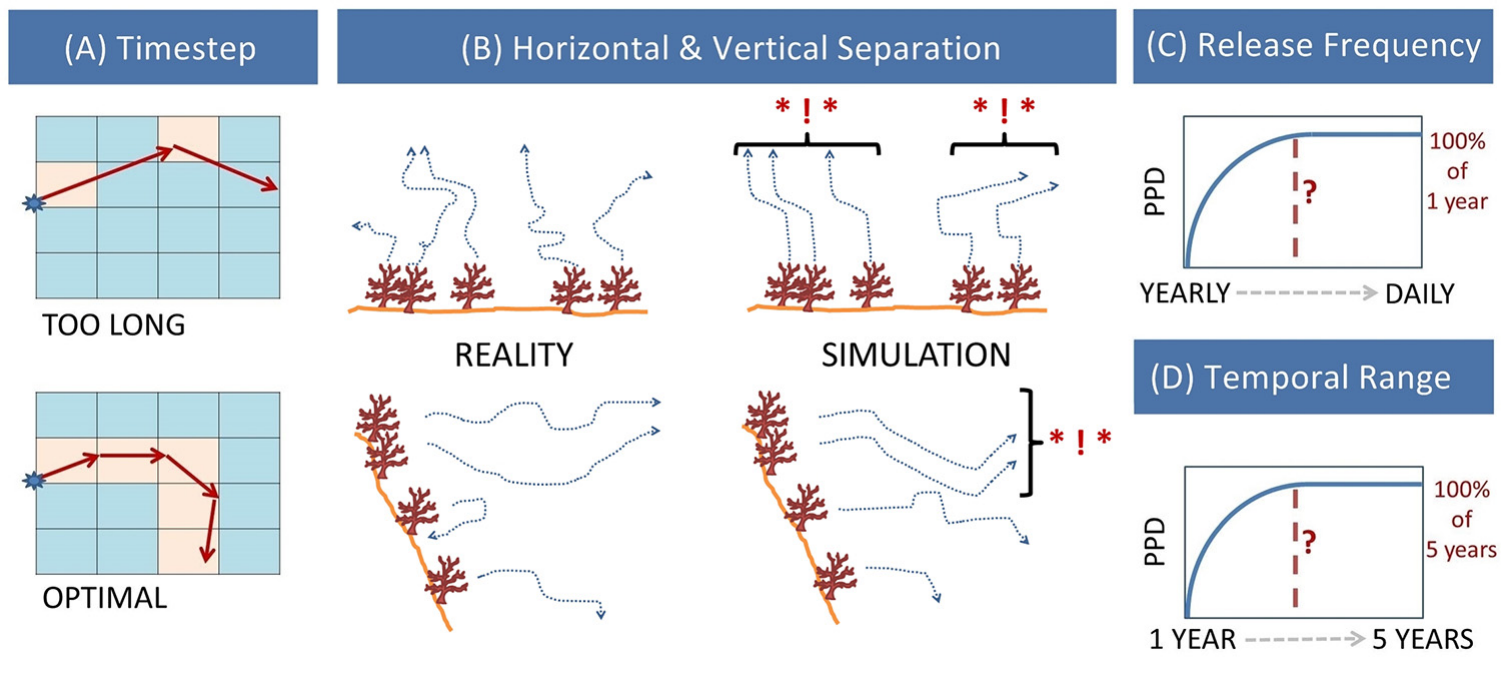
Descriptions of parameters tested in this study. (A) Timestep (TS) of the particle simulator governs how often the simulator asks for instruction. In this heavily simplified diagram, highlighted squares within the model grid have been asked for instruction–if the timestep is too long the resulting pathway may be very different from a timestep which is short enough to interrogate each grid square it encounters. (B) Horizontal (HS) and vertical separation (VS) of release points (spawning animals) in reality may be very small and yet result in different pathways of dispersal, but in a model simulation proximate release points may be spatially autocorrelated (here marked *! *) resulting in redundant simulations and a waste of computational power and analysis time. Both (C) release frequency (RF) and (D) temporal range (TR) tests act like a power analysis aiming to fulfil saturation of the models potential pathways of dispersal (PPD) either by simulating enough spawning events within a given period (e.g. daily within one year: release frequency), or by running simulations in enough years to be representative of a larger time period (e.g. five years: temporal range).

The TS of the particle simulator governs how often the simulator interrogates the hydrodynamic model for instruction to redirect the particle. This parameter is only dependent on the models used, and is not conditional upon the ecological question being studied. The aim here is to ensure the simulator asks for instruction frequently enough so that data is received from every grid cell along the dispersal pathway (as opposed to passing through several cells without “asking” for directions, Fig 2). As expected from the relationship between time, velocity and distance, TS is affected by the velocity range in the study area, and the resolution of the hydrodynamic model data (equivalent to distance). The Courant number (C) [39] is often used to test appropriate timesteps (ΔT) for a given grid resolution (ΔL) where average velocity ($\bar{V}$) is known:

$$C=\frac{\bar{V}\Delta T}{\Delta L}$$

This is a hydrodynamic modelling approach which can resolve questions about the TS [39], however an ecologist new to the world of hydrodynamic models may not be readily able to deduce average current speeds from model outputs, nor be able to identify the spatial and temporal range to average over. Should this be the case the test we offer here does not require prior knowledge of average velocity, but the results of the test can be used to back-compute average velocity. Ecologists who are not expert with NetCDF manipulation and Matlab may therefore benefit from this study’s method, and can cross-check their results using a *post hoc* Courant test. Whichever approach is used, ideally a TS test should be the first sensitivity test run to ensure that the results of all other tests are not affected by using the wrong default TS.

An ecologist may expect to define the HS of release points using realistic positioning of individual animals or by release area e.g. habitat patches such as reefs. However within a model proximate populations may be spatially autocorrelated even if they would not be considered as such in real life (Fig 2).For example, simulating larval release from individual animals, which may access different turbulences and micro currents in real life, could produce identical predicted pathways of dispersal (PPDs) for 500 proximate animals. Existing studies have recognised this and coarsened their HS, either by using repeated simulations of larval release from randomly chosen coordinates within a release area (e.g. [13]), or by using centroids of subdivided area polygons with a size defined by the resolution of the hydrodynamic model [5, 20, 38]. While the resolution of the model may provide a reasonable guide as an HS upper limit, the interpolant of the particle simulator further refines the combined model resolution and may allow release points with a sub-grid scale HS to produce independent PPDs. By defining the HS at which spatial autocorrelation is no longer a concern, decisions about adequately positioning release points can be better informed. HS should be expected to be dependent upon the horizontal resolution of the hydrodynamic model, the interpolative ability of the particle simulator, and the TS of the particle simulator. The planktonic larval duration (PLD, equivalent to the length of time the simulation is run) is also likely to have an effect as the longer you track particles, the more chance they have to deviate from each other.

The same issue applies to VS: whether spawning animals are situated on a slope or a vertical cliff, the combined model’s vertical resolution may affect the spatial autocorrelation of release points dispersed across depth bands (Fig 2). Simons et al. [20] tested this parameter between 2m and 30m from the surface, but in the deep sea there is much greater scope for varying sensitivity as the reference depth may be anywhere between 200m -11000m (i.e. from the edge of the continental slope to the bottom of an ocean trench).The vertical resolution of the model may vary with depth as it does when simulations are run within the native HYCOM model, or when running simulations based on model outputs with a stepped vertical structure (such as the previously mentioned Levitus convention). Results will likely be affected by the vertical data structure and the interpolative ability of the particle simulator. If vertical velocities and diffusion are included in model simulations, VS will also be affected by particle simulator TS.

For both the RF and TR tests we aim to assess model saturation and temporal autocorrelation. This is similar to a power analysis, for example defining how many quadrats would be required to represent the species composition of an area. In this case we search for the parameter values which maximise the potential of the models predictive power, aiming to find the coarsest resolution parameter setting which is still reflective of this asymptote. For the parameters tested this can be summarised as asking: how much temporal resolution can we lose while still adequately representing a high resolution baseline?

RF is akin to the number of spawning events in a given period of time (e.g. hourly, daily, etc.). Reality may define the spawning period (e.g. seasonal spawning may limit the simulation to a particular month), but the frequency of spawning events within that period is often unknown. Testing this parameter can offer a means to ensure that the maximum potential number of PPDs have been predicted whilst using the most computationally economic parameter setting (defining the point where the asymptote is reached, Fig 2). Equally if spawning periodicity is known (e.g. lunar periodicity [40] or annual planulation [41]), defining the point where RF reaches asymptote can show whether the model is capable of simulating your required setting, and whether there is a coarser setting which gives equivalent results. RF operates as a function of how temporally variable hydrodynamic conditions are within the model. If it is necessary to run a RF test, this should be done prior to HS and VS tests as it will affect whether you have captured the full variability of the modelled currents and therefore could affect the outcome of these tests. An inadequate RF is called an under-sampling/under-seeding problem [20, 42]. Other methods are available which offer similar results (e.g. [42]).

Ideally any modelling study will be representative of a longer period of time than actually simulated, for example Simons et al [20] used three years with different climatic extremes (El Niño/La Niña/normal) to encompass the maximal range of sensitivity and account for any chosen period of simulation in the study area. The TR test examines this sort of assumption by running a simulation over a longer period and checking whether any subset of years within this period (e.g. a set of three chosen based on climatic phenomena) could be deemed representative of the full simulation. In this test we especially aim to discover whether selecting years based on their North Atlantic Oscillation index (which would be a similar approach to Simons et al [20]) could give comparable results to running simulations over a longer period.

### Sensitivity tests

Release locations were defined based on HYCOM output topography: identifying sites which interface with ADS at each depth in order to simulate the release of benthic larvae. Dispersal simulations were run from 16 release locations equally spaced around the circumference of ADS at three different depths (700m, 1000m, 1500m) (Fig 1). The replicate 16 locations and three depths were used to control for differing states of hydrodynamic mixing. A planktonic larval duration (PLD) of 100days was used to capture the majority of known PLDs: Hilario et al [18] includes a study of known PLDs of eurybathic and deep-sea species stating that 50% would be accommodated by a PLD of 35d, and 75% by 69d, 100d equating to approximately 90% of species included in that study.

All sensitivity tests were carried out using multiple model runs with all parameters held the same throughout the test except for the parameter being permuted. All tests, unless otherwise stated, use: a particle tracking time-step of 1 hour, data from the year 2012 (4th Jan 2012 until 14th March 2013 to be inclusive of 100days tracking from 4th December 2012), the same 16 release positions per depth band at 700m, 1000m, and 1500m (see S1 File for exact release locations of each test), and a monthly RF as standard. Permuted increments for each test and custom setups which differ from the aforementioned standard are shown in (Table 1). HS increment locations were defined in ArcGIS 10.1 using buffers of appropriate radius centred on the baseline release locations, with final increment release locations placed along the seamount contour to maintain the interface with the seamount. All horizontal increments are subgrid-scale compared with the model resolution and are defined in degrees in order to be comparable to the model (projected distance e.g. kilometres would vary with latitude and be different in latitude vs longitude due to the model using grid cells defined in degrees). Standard baseline depths in the VS test were altered to best capture the different Levitus data resolutions. In Levitus, at 200m the next data point is 50m away, at 1000m it is 100m away, and at 1750m it is 250m away whereas at the standard depths used in other tests (700m, 1000m, 1500m) data points are all at the 100m resolution. The TR test was conducted over a five year period and all year combinations within this period compared (five 1 year, ten 2 year, ten 3 year, five 4 year, and one 5 year iterations). Year combinations were also assessed with respect to their North Atlantic Oscillation (NAO) state.

**Table 1:** Parameters tested in this study.

Sensitivity Test	Baseline (all increments compared to this)	Increment list		Customisation different from default
Timestep (TS)	*Individual spawning event* at default locations with TS = 1 hour.	3 hrs		n/a
		6 hrs		
		12 hrs		
		24 hrs		
Horizontal separation(HS)	*Individual spawning event* at default locations (= 0°)	+0.001°	Location modified by -	n/a
		+0.005°		
		+0.01°		
		+0.025°		
Vertical separation(VS)	*Individual spawning event* at modified standard depths (= 0m)	-0.1m	Depth modified by -	Depths were modified to monitor effect of Levitus structure (200m releases are above summit of seamount)
		-1m		
		-10m		
		-50m		
Release Frequency(RF)	*Multiple spawning events per individual location* from 365 releases (daily through 1 year)	183 releases (2 daily)		n/a
		104 releases (biweekly)		
		52 releases (weekly)		
		12 releases (monthly)		
		4 releases (seasonal)		
Temporal Range(TR)	*Multiple spawning events per individual location* from 5 years of releases (12 releases per year)	1 yr	Multiples are also permuted e.g. 3 yrs = year 1+year 3+year 5	n/a
		2 yrs		
		3 yrs		
		4 yrs		

### Analysis

There are two analysis techniques used in this study relating to the two methodological aims set out in the introduction (Fig 1).

#### Detecting spatial autocorrelation due to model structure

Each of the three parameters (HS, VS, and TS) was tested with a track-by-track comparison method in order to detect increment spatial autocorrelation or independence when compared with test baselines. One track was used as a baseline, and each increment track was compared to this over time using the curved earth distance separation between them as a measure of independence/autocorrelation (hereafter termed Distance Separation over Time (DST), Fig 1B). 16 different release locations, three depths and 12 times were used as replicates to provide a median averaged result which controls for different current regimes in space and time. There were therefore 576 baseline tracks tested against their corresponding four increment tracks (192 per baseline depth band), totalling 2880 particles simulated per baseline/increment pairing. Analysis was performed in Matlab with DST curved earth distances derived using the haversine equation. All analyses were based on median averaged results as compared to a reference 10km threshold. This threshold represents the distance below which tracks would be deemed spatially autocorrelated. This is an arbitrary threshold value which should be defined within the context of the study: in this case 10km was selected as an example due to Foster et al [5] and Paris et al [35] agreeing this as a distance where competent larvae are likely to be able to detect and orient towards suitable habitat.

Supplementary ANCOVA tests of increment and depth significance were run in the statistical software environment R. As spatial autocorrelation tests detect the patchiness of hydrodynamic velocity instruction, example velocity fields from HYCOM were also plotted using Matlab in order to provide further context to test results.

#### Assessing model saturation

The two parameters (RF and TR) analysed to assess model saturation could not be compared using a track-by-track comparison as they trial different temporal frequencies and therefore contain multiple tracks per baseline or increment. There are therefore 16 replicates per baseline depth band, or 48 replicates total. This amounts to a minimum and maximum number of particles simulated per test of RF: 192 (seasonal) / 17520 (daily) and TR: 576 (1 year)/2880 (5 years), with the maxima representing the baselines. The method used for this comparison is similar to that used by Simons et al [20]). The simulator outputs each particle’s position per day. These were converted into track lines in ArcGIS 10.1 and compiled into track density grids per baseline or increment. These grids were comprised of 2D spatial cells at half the resolution of the hydrodynamic model (~0.04°), with each cell displaying a count of the number of replicate tracks which pass through it. Track density plots differ from particle density distributions as no particle is counted twice per grid cell (representing numbers of tracks rather than repeated particle cell occupancy). The fraction of unexplained variance (FUV) was then found between each baseline/increment pairing: = 1 − *r*^2^, where r is the linear correlation coefficient between track density rasters, as compared on a cell by cell basis and summarised as a single value per raster pairing. Following Simons et al.’s [20] example, a 0.05 threshold FUV variance was used to define the point where variance in FUV was minimal. At this point the increment was interpreted as giving effectively the same result as the baseline.

## Results

### Spatial autocorrelation tests

**Fig 3:**
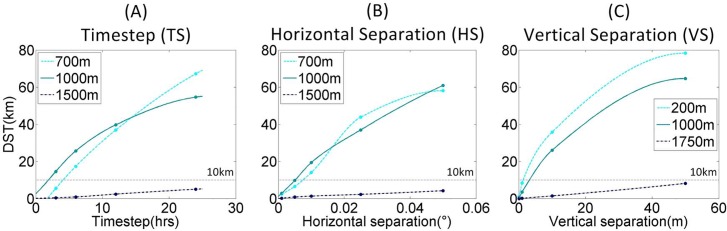
Results of spatial autocorrelation tests. Parameter increments from tests of (A) Timestep (TS), (B) Horizontal Separation (HS) and (C) Vertical Separation (VS), were plotted against median Distance Separation over Time (DST) with a piecewise polynomial interpolant applied between increment values of the same depth. A DST of 10km (based on records of larval habitat detection abilities) was taken as the autocorrelation/independence threshold allowing an optimal increment per depth to be derived from the intersection between the interpolant and the threshold. Optimal values derived from these plots are shown in Table 2.

**Fig 4:**
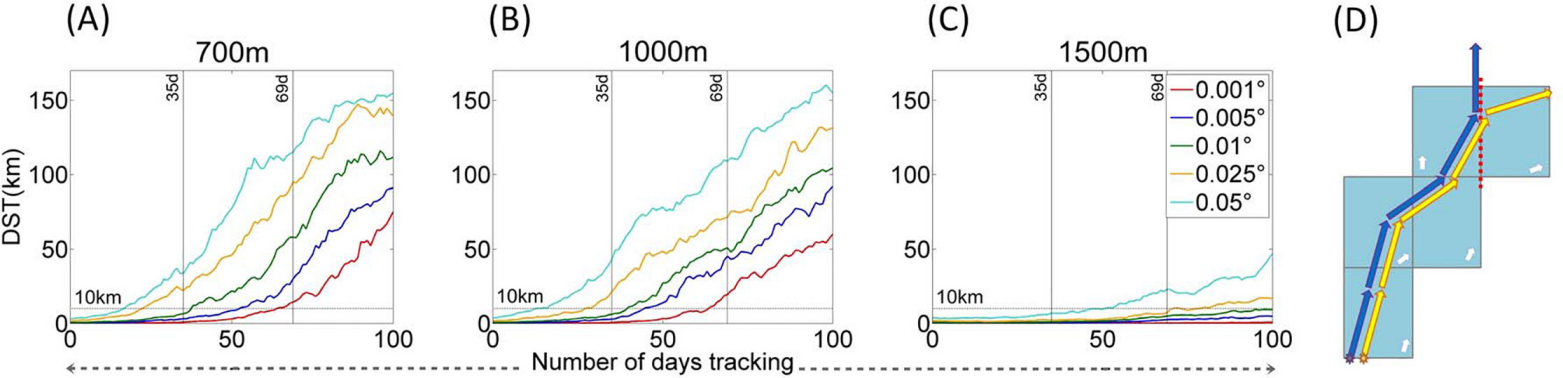
Plots, per depth, of Horizontal Separation (HS) increments Distance Separation over Time (DST) against tracking time (or Planktonic Larval Duration (PLD)). A 10km autocorrelation/independence threshold is shown and PLDs of 35days and 69days are marked reflecting PLDs which accommodate 50% and 75% of all known PLDs of deep-sea and eurybathic species (Hilario et al. [18]). Plots are shown for (A) 700m, (B) 1000m and (C) 1500m simulations. The right-hand diagram (D) demonstrates the possibility of two spatially autocorrelated tracks eventually accessing different instructions (represented by the white arrow in each grid cell) and deviating. This may account for the increased sensitivity over time, and may encourage interpretation as increased likelihood of error over time.

For all three tests, plots are shown of the median separation distance between each increment/baseline pairing (Fig 3). Results were averaged across all replicate locations and all days tracking and a piecewise cubic hermite interpolating polynomial line was fitted to the increment data per depth. These plots can be used to identify an increment value below which autocorrelation will occur. These values, hereafter referred to as ‘optimal values’, are shown in Table 2. S1 Fig includes boxplots of this data which are provided to give some scale of the variability in the data. This may be of use if, for example, rare dispersal events are important to the outcome of the study, or if suboptimal parameter values must be used and it is desirable to quantify the error that results. The results of ANCOVA tests of increment and depth significance can also be found in S1 File. Additional plots of median separation distance between each increment/baseline pairing over time (Fig 4) are shown for the HS test in order to show the effect of PLD on parameter sensitivity.

**Table 2:** Optimal value results of parameter sensitivity tests derived from plots in Fig 3 and Fig 5. All tests of spatial autocorrelation result in values less than the optimal value being spatially autocorrelated with the baseline (these tests define a high resolution baseline). All tests of model saturation (akin to a power analysis) result in values greater than the optimal value being temporally autocorrelated with the high resolution baseline.

Parameter	Test Type	Optimal Value	
Timestep (TS)	spatial autocorrelation	700m	4 hr
		1000m	2 hr
		1500m	48 hr (approx.)
Horizontal Separation (HS)	spatial autocorrelation	700m	0.0075°
		1000m	0.005°
		1500m	0.08° (approx.: this model resolution)
Vertical Separation (VS)	spatial autocorrelation	200m	1.5m
		1000m	3m
		1750m	60m
Release Frequency (RF)	model saturation	700m	150 releases per year
		1000m	160 releases per year
		1500m	75 releases per year
Temporal Range (TR)	model saturation	700m	4.3 yrs monthly releases
		1000m	4.3 yrs monthly releases
		1500m	4.1 yrs monthly releases

**Fig 5:**
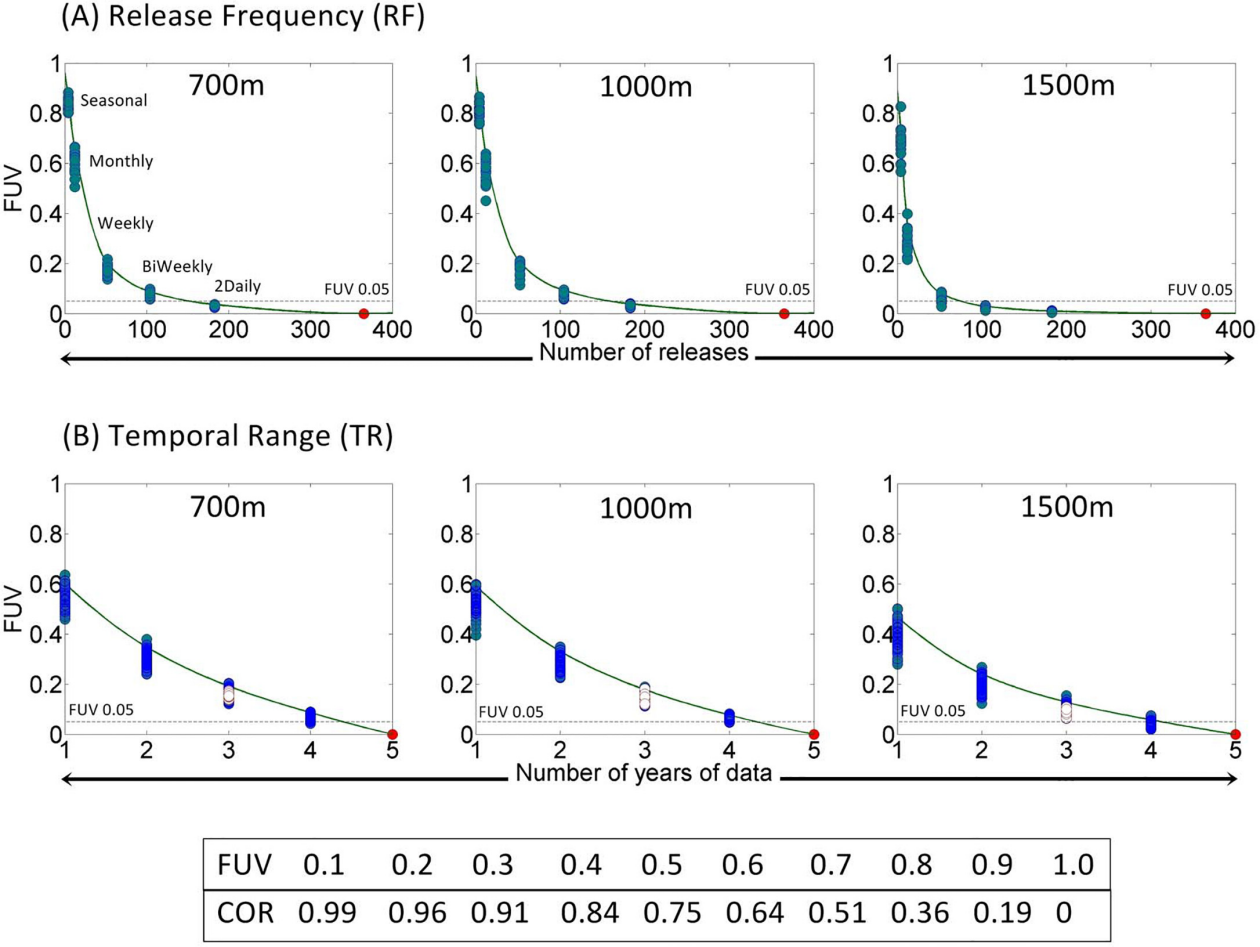
Results of temporal power analysis tests. Plots, per depth, for (A) Release Frequency (RF) and (B) Temporal Range (TR) tests, show increment values plotted against FUV scores. An FUV score represents one baseline/increment comparison, with a minimum of 16 replicates per increment (there are more in the temporal range test). A piecewise polynomial interpolant is fitted so that 95% of FUV scores fall below the line. The asymptotic threshold is defined as an FUV of 0.05 after Simons et al. [20]. Temporal range tests show FUV scores in white where three year datasets comprise years with optimal NAO indices (2009, 2010, and 2012 –see Fig 5). The table at the bottom shows the relationship between FUV and Pearson correlation values.

#### Horizontal separation

The plot in Fig 3 shows that DST increases with horizontal distance between release points. At 1500m depth, all tested increments were autocorrelated with a median DST well below the example 10km threshold. At 700m and 1000m depth, track deviance increased beyond the threshold at 0.0075° and 0.005° horizontal distance respectively providing a minimum distance for HS at these depths.

Fig 4 shows the effect of PLD (tracking time) upon HS sensitivity, with Hilario et al.’s [18] benchmark PLDs marked as examples. By looking at the median DST per day of tracking, per increment, it is clear that in tests with different PLDs the HS sensitivity would be different. In this test all increments of HS would remain autocorrelated (stay within the example 10km independence threshold distance) every day for up to 18 days at all depths. This means that with this model set up you cannot model PLDs of less than 18 days at sub-grid scale spacing without spatial autocorrelation. At 1500m all increments of HS are autocorrelated up to 52 days PLD. In order to model dispersal of species with a PLD of 35 days (Hilario et al.’s [18] heuristic PLD to encompass 50% of known deep-sea species) our results suggest that at 700m and 1000m depth >0.01° separation between horizontal release locations is required with 0.025° being the first tested increment that fulfils this criteria. At 1500m all increments trialled are spatially autocorrelated and thus a horizontal separation distance of >0.05° (potentially equivalent to model resolution 0.08°) is required. However, to model dispersal of species with a PLD of 69 days (Hilario et al.’s [18] heuristic PLD to encompass 75% of known deep-sea species) horizontal release locations of 0.001° would provide spatially independent larval dispersal pathways at 700m and 1000m. At 1500m >0.025° degrees separation between horizontal release locations is still required with 0.05° being the only tested increment that fulfils this criteria.

#### Vertical separation

Again all increments tested at the deepest baseline depth (1750m) were considered autocorrelated if using the 10km threshold (Fig 3) although the polynomial interpolation suggests that the threshold for independence may be approached at approximately 60m separation. Therefore it may be advisable to stratify release locations by 60m depth separation when at around 1750m depth. At 200m and 1000m baseline depths, VS is considerably more sensitive, with release locations separated by only 1.5m and 3m vertical distance respectively expected to track independently from each other.

#### Timestep

All TS tests were compared to a baseline of 1 hr. The first (3 hrs) increment at 1000m depth was already independent from the 1 hr track with the polynomial suggestive of a threshold at approximately 2hrs. It would therefore be advisable to use at least a 2hr timestep at 1000m depth. Interestingly the threshold, and therefore advised timestep, at 700m was closer to 4hrs (Fig 3). At 1500m depth the largest increment (24 hrs) still resulted in autocorrelated tracks when using the example 10km threshold. In spite of this it is advisable to stick with at least a daily frequency as the temporal resolution of the hydrodynamic data is also daily. Basic checks against the Courant number calculation suggest that these results are more conservative than an arbitrary area-averaged Courant number. E.g. at 1500m, average velocity over the NE Atlantic is 0.02m/s, giving a C = 0.46 when TS = 48 hr. Results were closer to using the maximum velocity in a Courant calculation over the trajectory-only domain. E.g. at 1500m, a maximum velocity of 0.05m/s was encountered by trajectories, giving C = 0.63 for a 24 hr TS, C only approaches convergence (C = 1) at 40 hrs (average velocity in this domain is 0.01m/s, C = 0.33 at 48 hrs).

#### Hydrodynamic Model Plots

**Fig 6:**
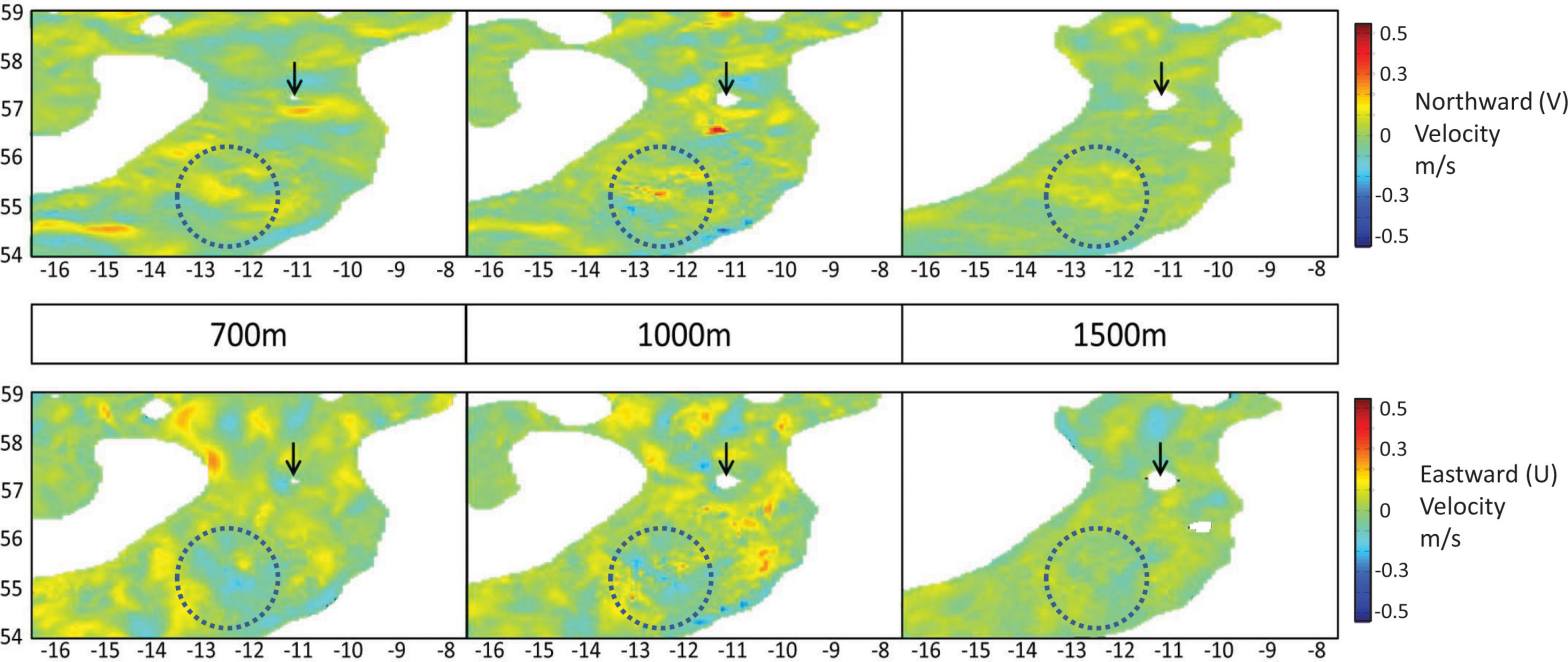
Example horizontal profiles of U and V velocity taken from one day in HYCOM (4^th^ Jan 2012). **HYCOM breaks down current velocities into directional components:** U velocity measures current speeds in an East (+ve)/West (-ve) direction (top three plots), and V in a North (+ve)/South (-ve) direction (bottom three plots). Anton Dohrn Seamount is marked in each depth slice with an arrow. Areas of different velocity from the background values appear as coloured patches. A comparison of areas within the dotted circles shows that profiles from 1000m have the greatest variation in current velocities (a greater number of small coloured patches). Profiles from 1500m show the least variation in velocity. Topographic contours are derived directly from HYCOM velocity data.

Matlab plots of the average velocity values for the standard 3 baseline depths are shown in Fig 6. The range of colours/velocity values in each plot shows that current velocities are more variable at 700m and 1000m than at 1500m. Closer inspection within the highlighted circles shows the 1000m depth slice as having the largest range in velocity values/colours, with patches of similar velocity covering smaller spatial extents than at the other two depths. These plots can be used to support.

### Model saturation tests

Each FUV value was plotted per increment and a piecewise cubic hermite interpolating polynomial line was fitted to the data in order that 95% of FUV values within each increment fell below it (Fig 5). This ensured that the polynomial was representative of the range of FUV values per increment and means that when the polynomial crosses the threshold 0.05 FUV the variance in FUV values should also have decreased below this value. The increment value where the polynomial crosses the 0.05 FUV threshold, hereafter referred to as the ‘optimal value’, is shown in Table 2.

#### Release Frequency

The FUV variance, or spread of points per increment, decreases steeply with the tested increment resolution (Fig 5). Seasonal releases do display little variance at 700m and 1000m, but have large FUV values indicating a correlation between maps of <0.36. The piecewise polynomial suggests that the FUV and 95% of its variance would decrease below the 0.05 threshold at 150–160 releases per year at 700m and 1000m. This result would mean that the track density plots derived from 150–160 releases in 2012 at these depths are effectively the same as track density plots from 365 releases in that year. At 1500m the variance in FUV values per increment is much higher, e.g. seasonal (four releases in a year) has a spread between 0.55 and 0.85 FUV (equivalent to a range of correlations from 0.70 to 0.28). At 1500m you would need at least 75 releases throughout the year to give an equivalent track dispersal plot to the daily release baseline.

#### Temporal Range

**Fig 7:**
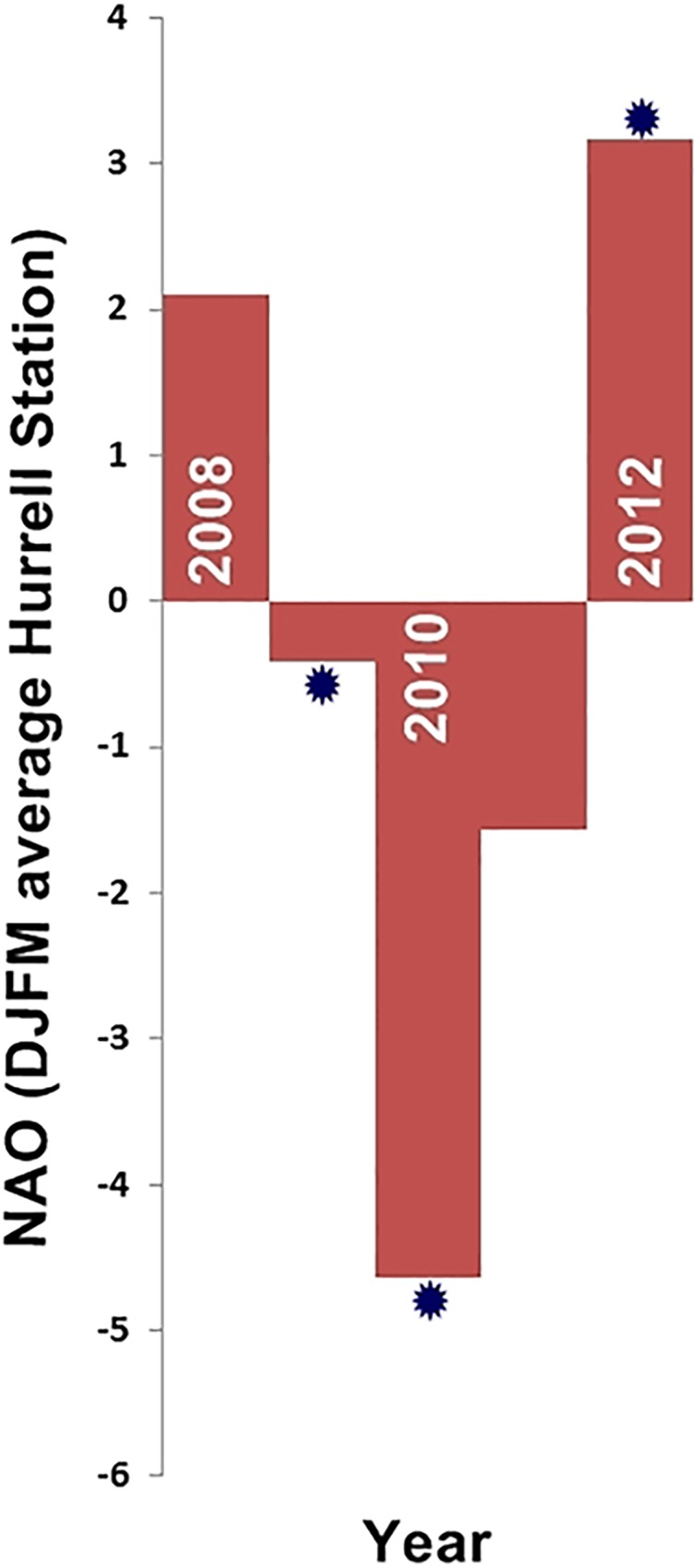
North Atlantic Oscillation (NAO) indices. Following Simons et al.’s [20] approach to El Niño, data from 2009 (NAO neutral), 2010 (strong negative) and 2012 (strong positive) should provide enough data to represent the full five years of simulations. Indices are plotted as December to March averages as recorded at Hurrell Station. Data sourced from http://climatedataguide.ucar.edu.

FUV decreases almost linearly with the number of years’ data when compared with the full 5 yr track density plot (Fig 5). The intersection of the piecewise polynomial with the 0.05 threshold suggests that 4.3 years of data would be required to represent the full five years of data at both 700m and 1000m, although approximately 4.1 years of data would be adequate at 1500m. If an approach similar to that of Simons et al [20] was used in this study only the three years starred in Fig 7 would be used, representing the two NAO extremes and a non-NAO event year, with results corresponding to the data points highlighted in white on Fig 5. Only at 1500m do these values approach the threshold FUV value, although they are still greater than 0.05. This result suggests that the three NAO states which may be selected as representative of a longer period could not be considered equivalent to the track density plot of a full five years of releases.

## Discussion

### Optimal values

The optimal values shown in Table 2 are appropriate for: this commonly employed pairing of models (HYCOM Global 1/12° and Connectivity Modeling System), in the Rockall Trough region of the Northeast Atlantic, for a generalised species with a PLD of 100 days and monthly spawning events. Should any of these conditions be altered, different optimal values may be found.

The values shown are considered optimal as exceeding optimal value resolution (i.e. reducing distance or timestep, or increasing frequency or number of years of simulation) may result in a waste of computational and analysis effort although all PPDs will be represented. Coarsening optimal value resolution (i.e. increasing distance or timestep, or decreasing frequency or number of years of simulation) could result in PPDs being omitted which the model was capable of predicting, potentially to the detriment of study conclusions.

While it is always preferable to use optimal values (or a higher resolution), sometimes it is not possible to achieve this and coarser values must be used. In this situation the results of sensitivity tests can offer a means to quantify error due to sub-optimal parameter values. The FUV method offers the best error quantification technique as FUV values are derived from linear correlations (r). When the 95% of FUV values variance control crosses the FUV = 0.05 threshold used in this study (RF and TS tests, after [20]), this means that 95% of baseline/increment comparison replicates exceeded a correlation of 0.9975. If the 0.05 threshold was not met, a correlation could be derived from the 95% interpolation line at the point of the highest resolution increment which can be used. For example, the RF test optimal value in this study was 150 releases per year at 1000m, but perhaps a weekly release frequency is the highest resolution setting possible. In Fig 5 the weekly (52 releases per year) increment corresponds with an FUV of ~0.2 at 700m and 1000m, and ~0.1 at 1500m. This value can be used to back-compute a correlation between the weekly and daily PPDs as

*FUV* = 1 − *r*^2^, so $r=\sqrt{\left(1-FUV\right)}$ (a table is provided at the bottom of Fig 5 to make estimating this even quicker). As the FUV is read from the polynomial interpolation this represents the FUV that 95% of replicate FUVs fall below. Therefore at 700m and 1000m the correlation between a weekly and daily release frequency is ~0.96 or greater (FUV 0.2), and at 1500m is ~0.99 or greater. This may help decide or at least report the adequacy of the sub-optimal parameter setting which must be used in place of, and compared to, the optimal setting. As all of these FUV calculations are based on Simons et al.’s heuristic 95% variance control value, there is also scope for varying the 0.05 FUV threshold value in line with study aims (something which is also discussed in Simons et al. [20]).

Error/accuracy is not so easily quantified using the described spatial autocorrelation technique, but median DST values of sub-optimal increments could be cited relative to the threshold, and boxplots of all DST values per increment (available in S1 Fig) can provide benchmarks in terms of DST quartiles and outlier ranges. For example, a study undertaken at around 1000m depth would ideally have a VS of ~3m based on a 10km threshold distance according to Fig 3. If the resolution of depth recordings requires VS to be 10m (a sub-optimal value), then the plot in Fig 3 shows that at 1000m a 10m VS should be expected to, on average (median), be separated from a baseline track by ~25km (2.5 times the distance of the optimal value). You can also tell from the box plots in S1 Fig that a VS of ~1m (closest to the optimal value of 2m) would have an upper quartile at around 100km, but at 10m VS the upper quartile approaches 250km (also ~2.5 times the optimal value). So you could estimate the accuracy of a 10m VS to be 2.5 times worse than that of the optimal value.

### Model Adequacy

As in Simons et al. [20], all tested parameters were affected by the strength of the mixing in the local system as portrayed within the hydrodynamic model. All tests can therefore provide insight into the hydrodynamic conditions within the chosen hydrodynamic model, and offer a means to ground-truth the combined hydrodynamic and simulator model’s abilities and limitations.

The suite of tests run in this study serve to capture different aspects of the model hydrodynamics with two tests (HS, VS) detecting spatial variance, and two (RF, TR) testing temporal variance, while TS interacts with both. The resolution of the hydrodynamic model may inform some of the combined model limitations, but adjustments need to be made for the interpolation provided by the particle simulator.

Our findings suggest that with increasing depth, fewer sub-grid release positions are required in order to represent the full range of dispersal pathways it is possible to simulate (when using this particular pairing of models in this region). For this to occur, neighbouring data points at shallower depths must produce steep differentials allowing different particle movement instructions to be obtained from interpolated intermediate locations. For example, neighbouring cells instructing 0.9m/s Northeast and 0.8m/s South, may result in an interpolation instructing 0.1m/s East-Southeast midway between data points. This therefore suggests a high spatial variability in hydrodynamic data.

However our results at depth (1500m, 1750m) were less sensitive. The HS test revealed no independence of tracks until HS approached model resolution, and the VS test recommended separation at ~ ¼ model resolution. This lack of sensitivity at depth implies weak differentials between vertically neighbouring cells. For example, neighbouring cells instructing 0.7m/s Northeast and 0.8m/s Northeast, may result in an interpolation instructing 0.75m/s Northeast midway between data points which is very similar to the neighbouring instructions.

Further evidence of this interpretation can be gained from the horizontal slices through the hydrodynamic data. Fig 6 shows three example horizontal slices through HYCOM, detailing the current velocities and their variability. It is clear from these plots that current velocities are more variable at 700m and 1000m than at 1500m. Furthermore the greatest variability in current velocities is found at 1000m, which accounts for the switch in sensitivity between these 700m and 1000m depths in HS and TS tests. The smaller velocity patches at 1000m require particles to travel smaller distances between interrogations to ensure receipt of every new potential instruction. This resulting in the smaller optimal HS and TS values at 1000m.

Now, with some idea of how the currents behave within the combined model, a comparison to empirical data can offer qualitative groundtruthing of model predictions and provide an assessment of combined model adequacy. HYCOM as a global model has been validated on a global scale but may not adequately represent the study area, so this is worth reviewing.

In this study the literature reveals that all shallower simulations undertaken in this area would occupy the same watermass—the poleward moving Eastern North Atlantic Water (ENAW). ENAW extends down to 1200m and characteristically exhibits mesoscale activity and relatively high current velocities [31, 43, 44]. This could account for this study’s predicted high variability of instruction through horizontal and vertical space at shallower depths. Winter convection in the area should also be expected to mix surface waters down to 600m typically, although this may extend to 1000m in severe winters [31].

Enhanced variation at 1000m may be due to a combination of factors. Eddies seen at shallower depths will have a smaller footprint at depth while the vorticity remains high, and the Hebrides Terrace Seamount summits at 1000m providing an additional stirring rod Southeast of ADS. There may also potentially be more interaction with intermediate water masses at 1000m. At this depth the core of Wyville Thomson ridge Overflow Water (WTOW) comes down from the north to the west of ADS, while Sub-Arctic Intermediate Water (SAIW) and Mediterranean Overflow Water (MOW) interact with the ENAW in a northward flow to the east of ADS [43].

As a result of this qualitative groundtruthing, the combined model in this study may be considered adequate when representing dominant water masses and mesoscale activity. However there is no obvious influence of SAIW or MOW in the model, so intermediate water masses probably remain largely un-parameterised (however the WTOW is visible in Fig 6 at both 700m and 1000m depths).

The results of the TS test can also be used to validate the model current speeds as the relationship velocity = distance/time can be related to the HS (distance) and TS (time) for each depth. For example, in this study:

- At 700m the recommended TS was 4 hrs (equivalent to ~500m/4 hrs based on 0.005° sensitivity) suggesting that local current speeds averaged around 0.4 m/s at this depth;
- The 1000m test recommends a maximum 2 hrs TS(750m/2 hrs based on 0.0075° sensitivity) equating to current speeds of 0.10m/s;
- The 1500m test may allow a TS >48 hr (8km/48 hrs based on 0.08° sensitivity) equivalent to maximum average current speeds of 0.046m/s (although this result is based on extending the polynomial interpolant far beyond the extent of the graph in Fig 3).

The literature does seem to bare out these predictions with Booth & Meldrum [45] recording currents with drifters (drogued between 66m and 166m) around Anton Dohrn as being up to 0.5m/s especially when caught in eddies, with a background flow of around 0.1m/s. Although derived from a different isopycnal model initialised from empirical data in the region, New & Smyth-Wright [46] estimate the Labrador Sea Water in the region (which only starts at 1500m) as ranging in current speeds from 0.004 m/s to 0.1 m/s, with some of the weakest of those current speeds recorded in the vicinity of ADS. ADS was the location of one of the observational transects in New & Smyth-Wright’s study [46] and these observations are in line with empirical observations reported in Ellet, Edwards & Bowers [47]).

Checks against the Courant number showed that this study’s TSs may be conservative, even when the velocity is averaged over only those grid cells encountered by trajectories. This is due to the 10km threshold introducing a maximum variance in result, while a Courant number could be based on an average over a large range of velocity values. Arguably a maximum velocity value could be used in Courant number calculations to approximate a similarly conservative result, but the method offered in this study has the benefit of inherently reflecting only the trajectory-encountered velocity values.

Both the RF and TR tests are representative of the variability in current velocities over time and can be used to assess this variability within the model. The RF result of 150–160 releases per year as equivalent to a daily release (at 700m and 1000m) suggest currents in this daily averaged model vary on the scale of roughly every two days. As tidal cycles are averaged out, this is representative of topographically induced mesoscale activity in the area. The TR result demonstrates high interannual variability in current velocities. This can be assessed against the North Atlantic Oscillation (NAO) data which is often attributed to driving large scale interannual hydrodynamic variability in the area due to its effect on convection regimes [46]. The test results show that the NAO dataset would not perform as well as the full test dataset if the 0.05 FUV variance threshold is deemed appropriate. Some literature agrees with this assessment, with NAO being linked to but not fully accounting for the interannual variability in the complex hydrodynamics of the Rockall Trough region [31, 43].This could have considerable consequence for the amount of data required to build PPDs valid over larger timescales, but at least the FUV and correlation scores can provide some quantitative estimate of how much is, or is not, captured within an NAO based dataset. As it stands using NAO selected years in this study would have a correlation to a five year baseline of approximately 0.92 (700m), 0.89 (1000m) and 0.95 (1500m) which may be considered adequate depending on the study premise.

This groundtruthing processes can inform the scenarios for future studies using this model set up; discerning whether the model set up should be used at all and further putting limits on the interpretations which can be drawn. The results of this study may suggest that HYCOM and CMS broadly agree with the observed hydrodynamics of the study area, but simulations cannot well represent rare dispersal events. Therefore all future studies using this model set up should be concerned with average PPDs and interpreted within this context. The lack of tides, sub-mesoscale, and even some mesoscale processes means that any results should be considered overestimates of dispersal abilities as the majority of these un-parameterised processes would have a retentive effect [48, 49]. Due to these inadequacies arguably the model would be better served as a statistical representation of dispersal probabilities rather than a deterministic model of larval fates.

The results of the HS over time test confirm that PLD will have an effect on positional sensitivity (Fig 4). These findings demonstrate the fact that autocorrelated tracks over time will eventually become free to deviate by accessing different instructions to the baseline (Fig 4, right-hand diagram). Therefore the longer the data is tracked, the more sensitive the parameter value becomes. This effect could be seen either as a need for a smaller separation distance when particles are tracked for longer, or as an increase in error with longer tracking times. Either way the result is informative as to how the model can be run and interpreted.

### Ecological and deep-sea consequences of these results

Although primarily representative of model performance, many of these results can be interpreted within an ecological context and may inform directions of future research.

Kough and Paris [50] recently undertook a study of spawning periodicity, akin to the RF test, and interpreted the results in terms of the ecological consequences of different spawning strategies. Spawning periodicity was found to control the number and persistence of reef network dispersal connections, with larval behaviour stabilising these connections. They conclude that spawning periodicity should be accurately included within biophysical models of larval dispersal due to its large potential impact on dispersal ability. In the instance where the RF cannot accurately be determined, as is likely especially in deep-sea ecology, this study offers a method of statistically predicting PPDs as opposed to the deterministic approach made possible with accurate information. The range of PPDs generated by undertaking sensitivity tests can provide potential maximum and minimum bounds of dispersal or be combined into a single probabilistic PPD. This way a useful prediction can still be made even when species specific data is lacking.

The TR test further supplements conclusions drawn by Kough and Paris [50], particularly in the event of seasonal spawners (which do also occur in the deep sea e.g. *Henricia lisa* (Clark 1949, Echinodermata) [41] or *Lophelia pertusa* (Linnaeus 1758, Cnidaria) [51]). The interannual variation in hydrodynamic conditions exemplified by the TR test shows the potential for change in PPDs over time. In which case the larvae of seasonal spawners may sometimes be released asynchronously, accessing different current patterns from previous cohorts. This may impact upon population persistence or potentially even drive speciation events [52].

Of importance to deep-sea ecological research is the effect of depth on parameter sensitivity. As mentioned previously this sensitivity can be linked to reduced current speeds and variability at depth (at least in this study area). This may mean that organisms accessing deeper currents have reduced potential dispersal abilities, and therefore rely upon stepping-stone like dispersal within larger metapopulations. While there is some evidence in support of this (e.g. abyssal bivalves, [53]) there is yet to be enough empirical data to ground-truth this theory. The effect of depth on parameter sensitivity also means that empirical positional data does not need to be of as high quality/resolution at depth, which may be a relief to deep-sea ecologists faced with, for example, the positional data of a trawl’s start and end points rather than a modern high resolution ROV location.

### Summary and Recommendations

This study was undertaken in order to better inform future work in the field of biophysical dispersal models and to enable more deep-sea ecologists to perform such modelling studies. To this end we supply the following step-by-step process, to summarise our advice on sensitivity tests, with case study examples shown to demonstrate the thought process.

1. Start Point
  You will have:
  - Already chosen a model set up (comprising of hydrodynamic model(s) & particle simulator).
  - Identified your study area.
  - Recognised there are parameters you need where the optimal value is unclear, and/or have recognised you are unaware of the models capabilities and limitations.
  - Planned the sort of ecological questions you wish to be asking to ensure that thresholds and parameter choice are suited to future work, including the tracking time/PLDs.
  - E.g. this study selected Hycom & CMS both of which are freely available and have previously been used in larval dispersal studies. Tests were performed in the Northeast Atlantic with the aim to pursue future work simulating passive larval release from benthic invertebrates within marine protected areas (MPAs) in the study area.
2. Identify parameters for sensitivity testing
  - It is worth performing sensitivity tests for as many parameters as possible, but if you need to prioritise then consider those where the optimal value is unclear, and at least select those which will test the modelled range of mixing strengths through space and time (i.e. representing x/y, depth, time) in your study area.
  - If biological individual based model parameters (e.g. behaviour) will be used in the final study, consider performing sensitivity tests on these also, especially where there is any uncertainty as to optimal values.
  - E.g. this study considers 5 parameters, including horizontal separation (x/y), vertical separation (depth), and release frequency/temporal range/timestep (time). All parameters affect the two most computationally intensive aspects of the simulation–the total number of particles being simulated, and the number of velocity fields being loaded into the simulator. Additional parameters worth testing in our case may include diffusivity values and subsequently the number of particles released per spawning event, these will be tested but are excluded here as they are specific to this particle simulator.
3. Identify the methods required for each parameter
  - This paper offers methods which can be used either where there are individual track baselines, or where there are multitrack baselines. Consider the impact of your research aims upon the methods you use e.g. will you be interested in average dispersal pathways or rare dispersal events?
  - In deep-sea studies your research may span a large depth range, if so be sure to stratify your testing in order to test for sensitivity differences with depth.
  - If multiple PLDs will be used consider retesting for each different tracking time
  - Consider what factors may affect each parameter and how they affect each other before designing your tests and order of testing.
  - E.g. our research will be interested in average dispersal pathways. Baseline tests were performed at 3 different depths which span the depth range included in future work. Aspects were considered such as the interaction between timestep and horizontal separation, and the impact of hydrodynamic model output structure on vertical separation. Tests against tracking time suggest recommendations will be different for different PLDs
4. Perform the tests and interpret the results
  - We recommend monitoring simulations (e.g. simulation time, record of memory usage) to gauge the parameter’s impact upon computational effort.
  - The results should help you define input parameter values, gain an understanding of how mixing occurs in your study area within your model, and gauge your capability to fulfil the full predictive power of your model setup.
  - At this point some preliminary groundtruthing can be performed in order to assess the adequacy of your model in your study area. Comparison to existing literature or datasets (e.g. Argo floats) may reveal why your model performs the way it does (e.g. water mass structures) and/or flag your model as inadequate, in which case you must start the process again with a new model setup.
    E.g. results in this case inform the structuring of release grids from specific sites (marine protected areas)–now with optimised values for horizontal and vertical separation of points. Should this result in too many release points (decided by computational power), multiple simulations can be run at shallower depths, using the maximum sub-optimal number of releases still possible, with release location randomly varied at a minimum distance of 0.005° from previous simulations. The effect of depth may recommend a stratification of simulations when performing ecological studies, with deeper MPAs requiring fewer (less separated) release points. Stratification will be informed by the watermass structure within the model. Timestep values did not greatly affect the time taken to run simulations so a timestep of one hour can be used throughout all future simulations. For species where no spawning periodicity is known, a release frequency per year will be set to weekly at a minimum (~90% correlation to a daily output), and will use at least three years spanning max/min/neutral NAO states (90% correlation to five years of simulated different NAO states).
5. Proceed to ecological studies using your model setup
  - You should now have a more intimate knowledge of the model setup workings and capabilities, allowing you to design your experiments appropriately and interpret your results responsibly.
    E.g. fortunately, as we are interested in average dispersal pathways this model setup should be adequate although this will not be proven until groundtruthed. Rare dispersal events will not be well represented especially at depth. Due to the lack of small scale hydrography represented, even in shallower water, results will likely be overestimates as sub-mesoscale and micro-scale hydrography would likely have promoted retention.
6. Repeat the process if the model set up or study area are changed
  - New model setups should be retested due to the effect of model resolution, structure, and strength and variability of modelled mixing, on the sensitivity of parameters.
  - As the strength of mixing in the study system (within the model) affects parameter sensitivity, different locations including different depths must be retested also.
    E.g. the results of this study are only suited to other dispersal research in the Rockall Trough region of the Northeast Atlantic using HYCOM and CMS ideally between 700m and 1500m (although some guidance is provided between 200m and 1750m due to the vertical separation test).

## Supporting Information

S1 Fig Boxplots accompanying timestep, horizontal separation, and vertical separation tests to provide a record of interquartile range and outliers per increment. This data may aid estimates of error if sub-optimal values must be selected.
(PDF)

S1 File List of release location coordinates and results of ANCOVA tests of increment and depth effects. (PDF)
