# Supplementary material for: Increasing the Depth of Current Understanding: Sensitivity Testing of Deep-Sea Larval Dispersal Models for Ecologists
Source: PLoS One. 2016 Aug 30;11(8):e0161220. doi: 10.1371/journal.pone.0161220 (PMC5004856; doi:10.1371/journal.pone.0161220)
Supplement: S1 Fig — This data may aid estimates of error if sub-optimal values must be selected. (PDF) [file pone.0161220.s001.pdf]

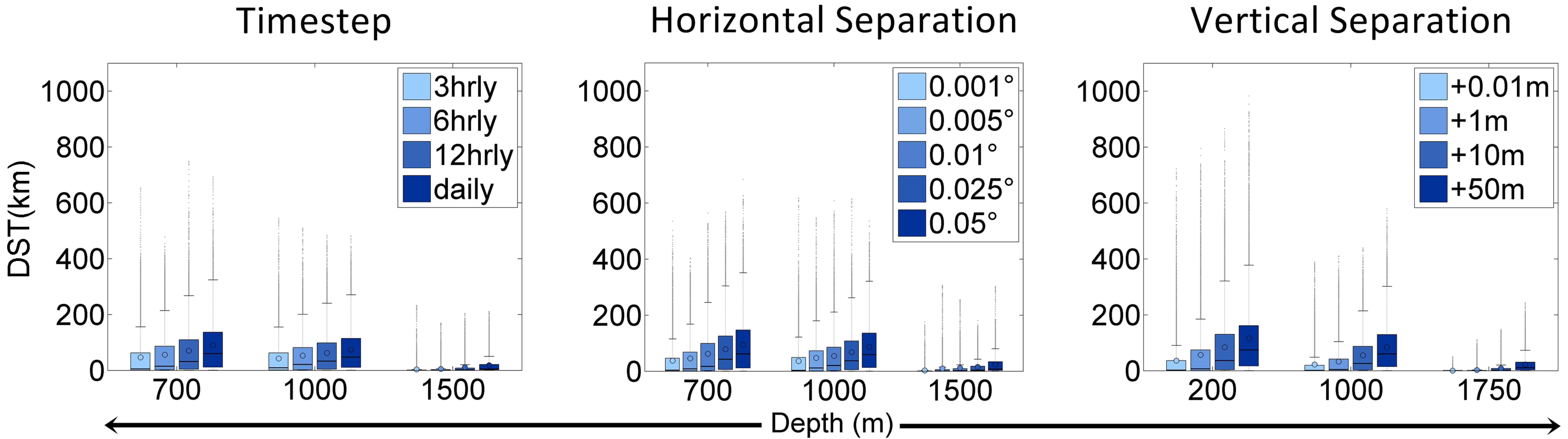

S1 Fig. Boxplots of timestep, horizontal separation, and vertical separation increment test medians, interquartile ranges, and outliers results. This data may aid estimates of error if sub-optimal values must be used.
