# Supplementary material for: Increasing the Depth of Current Understanding: Sensitivity Testing of Deep-Sea Larval Dispersal Models for Ecologists
Source: PLoS One. 2016 Aug 30;11(8):e0161220. doi: 10.1371/journal.pone.0161220 (PMC5004856; doi:10.1371/journal.pone.0161220)
Supplement: S1 File — (PDF) [file pone.0161220.s002.pdf]

Supplementary Material

(Accompanying MS Ross, Howell & Nimmo Smith "Increasing the *depth* of *current* understanding: Sensitivity testing of larval dispersal models for biologists")

Standard release positions

The following release positions were used as the baseline locations for the Timestep, Horizontal Positioning, Release Frequency, and Temporal Range tests (**Table S1**) and Vertical Positioning tests (**Table S2**). Vertical Positioning increments varied only in depth, not in lat lon, and were located based on the deepest increment contour in each depth test (250m, 1050m, 1800m). The increment release locations for Horizontal Positioning tests are available in **Table S3**.

Table S1 Baseline locations for all tests except the Vertical Positioning test

Depth	Posn #	Long	Lat	Depth	Posn #	Long	Lat	Depth	Posn #	Long	Lat
700	1	348.640	57.257	1000	1	348.480	57.257	1500	1	348.400	57.257
700	2	348.686	57.257	1000	2	348.560	57.301	1500	2	348.475	57.339
700	3	348.733	57.257	1000	3	348.640	57.344	1500	3	348.573	57.388
700	4	348.779	57.257	1000	4	348.730	57.339	1500	4	348.684	57.388
700	5	348.800	57.231	1000	5	348.810	57.295	1500	5	348.796	57.388
700	6	348.828	57.213	1000	6	348.890	57.251	1500	6	348.894	57.337
700	7	348.875	57.213	1000	7	348.970	57.207	1500	7	348.984	57.274
700	8	348.880	57.172	1000	8	349.030	57.164	1500	8	349.062	57.192
700	9	348.880	57.126	1000	9	348.960	57.113	1500	9	349.007	57.082
700	10	348.834	57.126	1000	10	348.910	57.053	1500	10	348.921	57.017
700	11	348.788	57.125	1000	11	348.820	57.038	1500	11	348.823	56.963
700	12	348.741	57.125	1000	12	348.740	57.004	1500	12	348.715	56.950
700	13	348.694	57.125	1000	13	348.650	56.994	1500	13	348.604	56.950
700	14	348.648	57.125	1000	14	348.570	57.034	1500	14	348.501	56.983
700	15	348.639	57.164	1000	15	348.490	57.078	1500	15	348.403	57.036
700	16	348.639	57.211	1000	16	348.480	57.166	1500	16	348.400	57.146

Table S2 Baseline locations for the Vertical Positioning test

Depth	Posn #	Long	Lat	Depth	Posn #	Long	Lat	Depth	Posn #	Long	Lat
200	1	348.64	57.257	1000	1	348.48	57.257	1750	1	348.344	57.282
200	2	348.686	57.257	1000	2	348.56	57.301	1750	2	348.428	57.383
200	3	348.733	57.257	1000	3	348.641	57.344	1750	3	348.57	57.437
200	4	348.779	57.257	1000	4	348.73	57.339	1750	4	348.684	57.437
200	5	348.8	57.231	1000	5	348.811	57.295	1750	5	348.799	57.432
200	6	348.828	57.213	1000	6	348.891	57.251	1750	6	348.914	57.391
200	7	348.875	57.213	1000	7	348.971	57.207	1750	7	349.056	57.321
200	8	348.88	57.172	1000	8	349.029	57.164	1750	8	349.165	57.191
200	9	348.88	57.126	1000	9	348.96	57.113	1750	9	349.099	57.02
200	10	348.834	57.126	1000	10	348.907	57.053	1750	10	348.977	56.954
200	11	348.788	57.125	1000	11	348.82	57.038	1750	11	348.822	56.929
200	12	348.741	57.125	1000	12	348.737	57.004	1750	12	348.712	56.93
200	13	348.694	57.125	1000	13	348.648	56.994	1750	13	348.6	56.929
200	14	348.648	57.125	1000	14	348.567	57.034	1750	14	348.459	56.954
200	15	348.639	57.164	1000	15	348.487	57.078	1750	15	348.335	57.024
200	16	348.639	57.211	1000	16	348.48	57.166	1750	16	348.334	57.146

Table S3 Increment locations for the Horizontal Positioning test

Increment		baseline		0.001		0.005		0.01		0.025	
Depth	Posn #	Long	Lat	Long	Lat	Long	Lat	Long	Lat	Long	Lat
700	1	348.640	57.257	348.879	57.126	348.875	57.126	348.870	57.126	348.855	57.126
700	2	348.686	57.257	348.833	57.126	348.829	57.126	348.824	57.126	348.809	57.126
700	3	348.733	57.257	348.787	57.125	348.783	57.125	348.778	57.125	348.763	57.125
700	4	348.779	57.257	348.740	57.125	348.736	57.125	348.731	57.125	348.716	57.125
700	5	348.800	57.231	348.693	57.125	348.689	57.125	348.684	57.125	348.669	57.125
700	6	348.828	57.213	348.648	57.126	348.647	57.130	348.646	57.135	348.642	57.149
700	7	348.875	57.213	348.639	57.165	348.639	57.169	348.639	57.174	348.639	57.189
700	8	348.880	57.172	348.639	57.212	348.639	57.216	348.639	57.221	348.640	57.236
700	9	348.880	57.126	348.641	57.257	348.645	57.257	348.650	57.257	348.665	57.257
700	10	348.834	57.126	348.687	57.257	348.691	57.257	348.696	57.257	348.711	57.257
700	11	348.788	57.125	348.734	57.257	348.738	57.257	348.743	57.257	348.758	57.257
700	12	348.741	57.125	348.780	57.256	348.782	57.253	348.785	57.249	348.795	57.238
700	13	348.694	57.125	348.801	57.230	348.804	57.228	348.808	57.226	348.821	57.217
700	14	348.648	57.125	348.829	57.213	348.833	57.213	348.838	57.213	348.853	57.213
700	15	348.639	57.164	348.875	57.212	348.876	57.208	348.876	57.203	348.878	57.188
700	16	348.639	57.211	348.880	57.171	348.880	57.167	348.880	57.162	348.880	57.147
1000	1	348.480	57.257	348.480	57.256	348.480	57.252	348.480	57.247	348.480	57.232
1000	2	348.560	57.301	348.559	57.301	348.556	57.299	348.551	57.296	348.538	57.289
1000	3	348.640	57.344	348.639	57.344	348.636	57.342	348.631	57.339	348.618	57.332
1000	4	348.730	57.339	348.729	57.339	348.725	57.339	348.720	57.340	348.705	57.340
1000	5	348.810	57.295	348.809	57.295	348.806	57.297	348.801	57.300	348.788	57.307
1000	6	348.890	57.251	348.889	57.251	348.886	57.253	348.881	57.256	348.868	57.263
1000	7	348.970	57.207	348.969	57.208	348.966	57.209	348.961	57.212	348.948	57.219
1000	8	349.030	57.164	349.029	57.164	349.026	57.167	349.022	57.170	349.010	57.178
1000	9	348.960	57.113	348.961	57.113	348.964	57.116	348.968	57.119	348.980	57.127
1000	10	348.910	57.053	348.911	57.054	348.913	57.057	348.916	57.061	348.926	57.072
1000	11	348.820	57.038	348.821	57.038	348.825	57.039	348.830	57.040	348.845	57.042
1000	12	348.740	57.004	348.741	57.004	348.745	57.006	348.749	57.008	348.763	57.014
1000	13	348.650	56.994	348.651	56.994	348.655	56.994	348.660	56.995	348.675	56.997
1000	14	348.570	57.034	348.571	57.033	348.575	57.032	348.579	57.030	348.592	57.023
1000	15	348.490	57.078	348.491	57.078	348.494	57.075	348.499	57.073	348.512	57.066
1000	16	348.480	57.166	348.480	57.165	348.481	57.161	348.481	57.156	348.483	57.141
1500	1	348.400	57.257	349.062	57.191	349.060	57.188	349.058	57.183	349.051	57.170
1500	2	348.475	57.339	349.006	57.081	349.003	57.079	348.999	57.076	348.987	57.067
1500	3	348.573	57.388	348.920	57.017	348.917	57.015	348.912	57.012	348.899	57.005
1500	4	348.684	57.388	348.822	56.963	348.818	56.962	348.813	56.962	348.798	56.960
1500	5	348.796	57.388	348.714	56.950	348.710	56.950	348.705	56.950	348.690	56.950
1500	6	348.894	57.337	348.603	56.950	348.599	56.952	348.594	56.953	348.580	56.958
1500	7	348.984	57.274	348.500	56.983	348.497	56.985	348.492	56.988	348.479	56.995
1500	8	349.062	57.192	348.403	57.037	348.403	57.041	348.403	57.046	348.402	57.061
1500	9	349.007	57.082	348.400	57.147	348.400	57.151	348.400	57.156	348.400	57.171
1500	10	348.921	57.017	348.401	57.258	348.403	57.261	348.407	57.264	348.417	57.275
1500	11	348.823	56.963	348.476	57.339	348.479	57.341	348.484	57.343	348.497	57.350
1500	12	348.715	56.950	348.574	57.388	348.578	57.388	348.583	57.388	348.598	57.388
1500	13	348.604	56.950	348.685	57.388	348.689	57.388	348.694	57.388	348.709	57.388
1500	14	348.501	56.983	348.797	57.388	348.800	57.386	348.805	57.383	348.818	57.376
1500	15	348.403	57.036	348.895	57.336	348.898	57.334	348.902	57.331	348.914	57.323
1500	16	348.400	57.146	348.985	57.273	348.987	57.270	348.991	57.267	349.001	57.256

ANCOVA tests of increment and depth effect

The following GLMs were performed as ANCOVA tests of significance, confirming that the tested increments were having an effect on the straight line distance (sld) from baseline, and that depth also had an effect in each of the track dispersion technique tests.

The GLMs were performed in R, with increment (incr), depth, and day included in ANCOVAs where possible as a factor variables (different inclusions were trialled, vertical separation test required exclusion of factor(depth) due to collinearity with increment).

Post-hoc Tukey tests of factor (incr) and factor (depth) show the inter-relationship of factor levels and their respective significance.

“drop1” summaries are shown by way of summarising all the levels of a factor into an individual p value.

All tests resulted in the best GLM when straight line distance (sld) was included as square root transformed (sqrtsld).

TIMESTEP

```
> summary(M2)

Call:
glm(formula = sqrtsld ~ factor(incr)  + factor(depth)  + factor(day), fami
ly = Gamma,
    data = m2)

Deviance Residuals:
    Min       1Q   Median       3Q      Max
-2.3011  -0.7126  -0.1961   0.3128   2.6368

Coefficients:
                 Estimate Std. Error t value Pr(>|t|)
(Intercept)     1.4153516  0.0227323  62.262   <2e-16 ***
factor(incr) 21600     -0.0360491  0.0009790 -36.823   <2e-16 ***
factor(incr) 43200     -0.0644112  0.0009073 -70.995   <2e-16 ***
factor(incr) 86400     -0.0849766  0.0008628 -98.493   <2e-16 ***
factor(depth) 1000        0.0044278  0.0005232   8.462   <2e-16 ***
factor(depth) 1500        0.2634405  0.0012699 207.449   <2e-16 ***
factor(day)2   -0.2556197  0.0293241  -8.717   <2e-16 ***
factor(day)3   -0.3639115  0.0282276 -12.892   <2e-16 ***
factor(day)4   -0.4546708  0.0274111 -16.587   <2e-16 ***
factor(day)5   -0.5285107  0.0267660 -19.746   <2e-16 ***
factor(day)6   -0.5947293  0.0262091 -22.692   <2e-16 ***
factor(day)7   -0.6554506  0.0257520 -25.452   <2e-16 ***
factor(day)8   -0.7117822  0.0253333 -28.097   <2e-16 ***
factor(day)9   -0.7461221  0.0250736 -29.757   <2e-16 ***
factor(day)10  -0.7812083  0.0248419 -31.447   <2e-16 ***
factor(day)11  -0.8062077  0.0246781 -32.669   <2e-16 ***
factor(day)12  -0.8272296  0.0245420 -33.707   <2e-16 ***
factor(day)13  -0.8483648  0.0244178 -34.744   <2e-16 ***
factor(day)14  -0.8734081  0.0242695 -35.988   <2e-16 ***
factor(day)15  -0.8925951  0.0241652 -36.937   <2e-16 ***
factor(day)16  -0.9086135  0.0240730 -37.744   <2e-16 ***
factor(day)17  -0.9207697  0.0240060 -38.356   <2e-16 ***
factor(day)18  -0.9356316  0.0239296 -39.099   <2e-16 ***
factor(day)19  -0.9552016  0.0238305 -40.083   <2e-16 ***
factor(day)20  -0.9686523  0.0237634 -40.762   <2e-16 ***
factor(day)21  -0.9803764  0.0236999 -41.366   <2e-16 ***
factor(day)22  -0.9935839  0.0236429 -42.025   <2e-16 ***
factor(day)23  -1.0112543  0.0235628 -42.917   <2e-16 ***
factor(day)24  -1.0272939  0.0234938 -43.726   <2e-16 ***
factor(day)25  -1.0373365  0.0234504 -44.235   <2e-16 ***
factor(day)26  -1.0465864  0.0234116 -44.704   <2e-16 ***
factor(day)27  -1.0575608  0.0233681 -45.257   <2e-16 ***
factor(day)28  -1.0671806  0.0233295 -45.744   <2e-16 ***
factor(day)29  -1.0774290  0.0232919 -46.258   <2e-16 ***
factor(day)30  -1.0862412  0.0232579 -46.704   <2e-16 ***
factor(day)31  -1.0943747  0.0232283 -47.114   <2e-16 ***
factor(day)32  -1.1023334  0.0232011 -47.512   <2e-16 ***
factor(day)33  -1.1095071  0.0231772 -47.871   <2e-16 ***
factor(day)34  -1.1162006  0.0231547 -48.206   <2e-16 ***
factor(day)35  -1.1228262  0.0231314 -48.541   <2e-16 ***
factor(day)36  -1.1302602  0.0231076 -48.913   <2e-16 ***
factor(day)37  -1.1378160  0.0230838 -49.291   <2e-16 ***
factor(day)38  -1.1463744  0.0230584 -49.716   <2e-16 ***
factor(day)39  -1.1528995  0.0230391 -50.041   <2e-16 ***
factor(day)40  -1.1582806  0.0230233 -50.309   <2e-16 ***
factor(day)41  -1.1633059  0.0230091 -50.559   <2e-16 ***
factor(day)42  -1.1678536  0.0229962 -50.785   <2e-16 ***
factor(day)43  -1.1725445  0.0229834 -51.017   <2e-16 ***
factor(day)44  -1.1768159  0.0229719 -51.228   <2e-16 ***
factor(day)45  -1.1809410  0.0229613 -51.432   <2e-16 ***
factor(day)46  -1.1847969  0.0229515 -51.622   <2e-16 ***
factor(day)47  -1.1888055  0.0229413 -51.819   <2e-16 ***
factor(day)48  -1.1926098  0.0229316 -52.007   <2e-16 ***
factor(day)49  -1.1971475  0.0229212 -52.229   <2e-16 ***
```

```
factor(day)50  -1.2002038  0.0229134 -52.380   <2e-16 ***
factor(day)51  -1.2036305  0.0229056 -52.548   <2e-16 ***
factor(day)52  -1.2060171  0.0228998 -52.665   <2e-16 ***
factor(day)53  -1.2095028  0.0228919 -52.835   <2e-16 ***
factor(day)54  -1.2129432  0.0228842 -53.003   <2e-16 ***
factor(day)55  -1.2161284  0.0228772 -53.159   <2e-16 ***
factor(day)56  -1.2190321  0.0228710 -53.300   <2e-16 ***
factor(day)57  -1.2214450  0.0228660 -53.418   <2e-16 ***
factor(day)58  -1.2237505  0.0228611 -53.530   <2e-16 ***
factor(day)59  -1.2259941  0.0228564 -53.639   <2e-16 ***
factor(day)60  -1.2278951  0.0228525 -53.731   <2e-16 ***
factor(day)61  -1.2297690  0.0228488 -53.822   <2e-16 ***
factor(day)62  -1.2317148  0.0228449 -53.916   <2e-16 ***
factor(day)63  -1.2335782  0.0228412 -54.007   <2e-16 ***
factor(day)64  -1.2354386  0.0228377 -54.097   <2e-16 ***
factor(day)65  -1.2370878  0.0228345 -54.176   <2e-16 ***
factor(day)66  -1.2388519  0.0228312 -54.261   <2e-16 ***
factor(day)67  -1.2403546  0.0228283 -54.334   <2e-16 ***
factor(day)68  -1.2418853  0.0228255 -54.408   <2e-16 ***
factor(day)69  -1.2435735  0.0228224 -54.489   <2e-16 ***
factor(day)70  -1.2451845  0.0228195 -54.567   <2e-16 ***
factor(day)71  -1.2467598  0.0228167 -54.643   <2e-16 ***
factor(day)72  -1.2484741  0.0228136 -54.725   <2e-16 ***
factor(day)73  -1.2501990  0.0228106 -54.808   <2e-16 ***
factor(day)74  -1.2517625  0.0228079 -54.883   <2e-16 ***
factor(day)75  -1.2530891  0.0228057 -54.946   <2e-16 ***
factor(day)76  -1.2541738  0.0228039 -54.998   <2e-16 ***
factor(day)77  -1.2554087  0.0228018 -55.057   <2e-16 ***
factor(day)78  -1.2566027  0.0227998 -55.115   <2e-16 ***
factor(day)79  -1.2577496  0.0227979 -55.169   <2e-16 ***
factor(day)80  -1.2590500  0.0227958 -55.232   <2e-16 ***
factor(day)81  -1.2602485  0.0227939 -55.289   <2e-16 ***
factor(day)82  -1.2612693  0.0227923 -55.337   <2e-16 ***
factor(day)83  -1.2621679  0.0227909 -55.380   <2e-16 ***
factor(day)84  -1.2631172  0.0227894 -55.426   <2e-16 ***
factor(day)85  -1.2639498  0.0227881 -55.465   <2e-16 ***
factor(day)86  -1.2648473  0.0227867 -55.508   <2e-16 ***
factor(day)87  -1.2657073  0.0227854 -55.549   <2e-16 ***
factor(day)88  -1.2666241  0.0227840 -55.593   <2e-16 ***
factor(day)89  -1.2674879  0.0227827 -55.634   <2e-16 ***
factor(day)90  -1.2682465  0.0227816 -55.670   <2e-16 ***
factor(day)91  -1.2688751  0.0227807 -55.700   <2e-16 ***
factor(day)92  -1.2695793  0.0227796 -55.733   <2e-16 ***
factor(day)93  -1.2702604  0.0227786 -55.765   <2e-16 ***
factor(day)94  -1.2709903  0.0227776 -55.800   <2e-16 ***
factor(day)95  -1.2717404  0.0227765 -55.836   <2e-16 ***
factor(day)96  -1.2725325  0.0227754 -55.873   <2e-16 ***
factor(day)97  -1.2730863  0.0227746 -55.899   <2e-16 ***
factor(day)98  -1.2735419  0.0227739 -55.921   <2e-16 ***
factor(day)99  -1.2740967  0.0227732 -55.947   <2e-16 ***
factor(day)100 -1.2746975  0.0227722 -55.976   <2e-16 ***
---
Signif. codes:  0 ‘***’ 0.001 ‘**’ 0.01 ‘*’ 0.05 ‘.’ 0.1 ‘ ’ 1

(Dispersion parameter for Gamma family taken to be 0.5478042)

    Null deviance: 250032  on 228563  degrees of freedom
Residual deviance: 124803  on 228459  degrees of freedom
AIC: 1000935

Number of Fisher Scoring iterations: 6


> drop1(M2, test = "Chi") # for whether to drop one also for p value of ma
in effect of factor Chi for GLM F for lm (GAM use GAM summary)
Single term deletions

Model:
sqrtsld ~ factor(incr)  + factor(depth)  + factor(day)
```

```
            Df Deviance     AIC scaled dev.  Pr(>Chi)
<none>           124803 1000935
factor(incr)        3   131767 1013641       12712 < 2.2e-16 ***
factor(depth)          2   164921 1074166      73235 < 2.2e-16 ***
factor(day) 99   192834 1124926      124189 < 2.2e-16 ***
---
Signif. codes:  0 ‘***’ 0.001 ‘**’ 0.01 ‘*’ 0.05 ‘.’ 0.1 ‘ ’ 1


> posth <- glht(M2, linfct=mcp(factor(incr) ="Tukey"), data = m2)
> summary(posth)

         Simultaneous Tests for General Linear Hypotheses

Multiple Comparisons of Means: Tukey Contrasts


Fit: glm(formula = sqrtsld ~ factor(incr)  + factor(depth)  + factor(day),
 family = Gamma,
    data = m2)

Linear Hypotheses:
                     Estimate Std. Error z value Pr(>|z|)
21600 - 10800 == 0 -0.0360491  0.0009790  -36.82   <2e-16 ***
43200 - 10800 == 0 -0.0644112  0.0009073  -71.00   <2e-16 ***
86400 - 10800 == 0 -0.0849766  0.0008628  -98.49   <2e-16 ***
43200 - 21600 == 0 -0.0283620  0.0007804  -36.34   <2e-16 ***
86400 - 21600 == 0 -0.0489275  0.0007266  -67.33   <2e-16 ***
86400 - 43200 == 0 -0.0205654  0.0006207  -33.13   <2e-16 ***
---
Signif. codes:  0 ‘***’ 0.001 ‘**’ 0.01 ‘*’ 0.05 ‘.’ 0.1 ‘ ’ 1
(Adjusted p values reported -- single-step method)

> posth2 <- glht(M2, linfct=mcp(factor(depth) ="Tukey"), data = m2)
> summary(posth2)

         Simultaneous Tests for General Linear Hypotheses

Multiple Comparisons of Means: Tukey Contrasts


Fit: glm(formula = sqrtsld ~ factor(incr)  + factor(depth)  + factor(day),
 family = Gamma,
    data = m2)

Linear Hypotheses:
                  Estimate Std. Error z value Pr(>|z|)
1000 - 700 == 0  0.0044278  0.0005232   8.462   <2e-16 ***
1500 - 700 == 0  0.2634405  0.0012699 207.449   <2e-16 ***
1500 - 1000 == 0 0.2590127  0.0012738 203.338   <2e-16 ***
---
Signif. codes:  0 ‘***’ 0.001 ‘**’ 0.01 ‘*’ 0.05 ‘.’ 0.1 ‘ ’ 1
(Adjusted p values reported -- single-step method)
```

HORIZONTAL

```
> summary(M2)

Call:
glm(formula = sqrtsld ~ factor(incr) + factor(depth) + factor(day),
    family = Gamma, data = m2)

Deviance Residuals:
    Min       1Q   Median       3Q      Max
-2.4419  -0.7769  -0.1865   0.3349   2.6224

Coefficients:
                    Estimate Std. Error t value Pr(>|t|)
(Intercept)        1.2403407  0.0200845  61.756  < 2e-16 ***
factor(incr)0.005 -0.0567097  0.0010646 -53.270  < 2e-16 ***
factor(incr)0.01  -0.0761433  0.0010140 -75.094  < 2e-16 ***
factor(incr)0.025 -0.0887582  0.0009907 -89.588  < 2e-16 ***
factor(depth)1000  0.0025041  0.0005788   4.326 1.52e-05 ***
factor(depth)1500  0.2393491  0.0014968 159.908  < 2e-16 ***
factor(day)1      -0.1372091  0.0269471  -5.092 3.55e-07 ***
factor(day)2      -0.0898570  0.0283419  -3.170  0.00152 **
factor(day)3      -0.1340915  0.0278430  -4.816 1.47e-06 ***
factor(day)4      -0.1951726  0.0271220  -7.196 6.22e-13 ***
factor(day)5      -0.2416328  0.0265915  -9.087  < 2e-16 ***
factor(day)6      -0.2803066  0.0261249 -10.729  < 2e-16 ***
factor(day)7      -0.3313541  0.0255574 -12.965  < 2e-16 ***
factor(day)8      -0.3882098  0.0249876 -15.536  < 2e-16 ***
factor(day)9      -0.4233234  0.0245957 -17.211  < 2e-16 ***
factor(day)10     -0.4650484  0.0241990 -19.218  < 2e-16 ***
factor(day)11     -0.5006132  0.0238305 -21.007  < 2e-16 ***
factor(day)12     -0.5329773  0.0235295 -22.651  < 2e-16 ***
factor(day)13     -0.5584487  0.0232809 -23.987  < 2e-16 ***
factor(day)14     -0.5879713  0.0230068 -25.556  < 2e-16 ***
factor(day)15     -0.6138355  0.0227722 -26.955  < 2e-16 ***
factor(day)16     -0.6396403  0.0225485 -28.367  < 2e-16 ***
factor(day)17     -0.6680682  0.0223314 -29.916  < 2e-16 ***
factor(day)18     -0.6872117  0.0221735 -30.992  < 2e-16 ***
factor(day)19     -0.7077957  0.0220174 -32.147  < 2e-16 ***
factor(day)20     -0.7307176  0.0218550 -33.435  < 2e-16 ***
factor(day)21     -0.7511900  0.0217073 -34.605  < 2e-16 ***
factor(day)22     -0.7708832  0.0215687 -35.741  < 2e-16 ***
factor(day)23     -0.7905628  0.0214357 -36.881  < 2e-16 ***
factor(day)24     -0.8103983  0.0213101 -38.029  < 2e-16 ***
factor(day)25     -0.8241237  0.0212230 -38.832  < 2e-16 ***
factor(day)26     -0.8392388  0.0211329 -39.712  < 2e-16 ***
factor(day)27     -0.8519978  0.0210586 -40.458  < 2e-16 ***
factor(day)28     -0.8655583  0.0209855 -41.246  < 2e-16 ***
factor(day)29     -0.8760881  0.0209219 -41.874  < 2e-16 ***
factor(day)30     -0.8885824  0.0208553 -42.607  < 2e-16 ***
factor(day)31     -0.8995508  0.0208006 -43.246  < 2e-16 ***
factor(day)32     -0.9102424  0.0207497 -43.868  < 2e-16 ***
factor(day)33     -0.9186687  0.0207072 -44.365  < 2e-16 ***
factor(day)34     -0.9264198  0.0206725 -44.814  < 2e-16 ***
factor(day)35     -0.9328998  0.0206421 -45.194  < 2e-16 ***
factor(day)36     -0.9387058  0.0206155 -45.534  < 2e-16 ***
factor(day)37     -0.9457545  0.0205849 -45.944  < 2e-16 ***
factor(day)38     -0.9531626  0.0205540 -46.373  < 2e-16 ***
factor(day)39     -0.9594909  0.0205273 -46.742  < 2e-16 ***
factor(day)40     -0.9659562  0.0205021 -47.115  < 2e-16 ***
factor(day)41     -0.9705591  0.0204827 -47.384  < 2e-16 ***
factor(day)42     -0.9769490  0.0204592 -47.751  < 2e-16 ***
factor(day)43     -0.9820915  0.0204395 -48.049  < 2e-16 ***
factor(day)44     -0.9866868  0.0204224 -48.314  < 2e-16 ***
factor(day)45     -0.9912714  0.0204069 -48.575  < 2e-16 ***
factor(day)46     -0.9948465  0.0203931 -48.783  < 2e-16 ***
factor(day)47     -0.9996330  0.0203773 -49.056  < 2e-16 ***
factor(day)48     -1.0040972  0.0203620 -49.312  < 2e-16 ***
factor(day)49     -1.0087442  0.0203469 -49.577  < 2e-16 ***
factor(day)50     -1.0126802  0.0203343 -49.802  < 2e-16 ***
```

```
factor(day)51     -1.0159710  0.0203231 -49.991  < 2e-16 ***
factor(day)52     -1.0193557  0.0203129 -50.183  < 2e-16 ***
factor(day)53     -1.0227211  0.0203030 -50.373  < 2e-16 ***
factor(day)54     -1.0251749  0.0202951 -50.513  < 2e-16 ***
factor(day)55     -1.0277357  0.0202875 -50.659  < 2e-16 ***
factor(day)56     -1.0302478  0.0202800 -50.801  < 2e-16 ***
factor(day)57     -1.0326331  0.0202732 -50.936  < 2e-16 ***
factor(day)58     -1.0349703  0.0202665 -51.068  < 2e-16 ***
factor(day)59     -1.0367660  0.0202610 -51.170  < 2e-16 ***
factor(day)60     -1.0393773  0.0202543 -51.316  < 2e-16 ***
factor(day)61     -1.0413156  0.0202488 -51.426  < 2e-16 ***
factor(day)62     -1.0436444  0.0202425 -51.557  < 2e-16 ***
factor(day)63     -1.0459105  0.0202363 -51.685  < 2e-16 ***
factor(day)64     -1.0477384  0.0202316 -51.787  < 2e-16 ***
factor(day)65     -1.0492598  0.0202275 -51.873  < 2e-16 ***
factor(day)66     -1.0508117  0.0202233 -51.960  < 2e-16 ***
factor(day)67     -1.0527463  0.0202186 -52.068  < 2e-16 ***
factor(day)68     -1.0547534  0.0202138 -52.180  < 2e-16 ***
factor(day)69     -1.0562203  0.0202100 -52.262  < 2e-16 ***
factor(day)70     -1.0580920  0.0202055 -52.367  < 2e-16 ***
factor(day)71     -1.0599845  0.0202009 -52.472  < 2e-16 ***
factor(day)72     -1.0619819  0.0201964 -52.583  < 2e-16 ***
factor(day)73     -1.0634689  0.0201928 -52.666  < 2e-16 ***
factor(day)74     -1.0649568  0.0201894 -52.748  < 2e-16 ***
factor(day)75     -1.0660806  0.0201868 -52.811  < 2e-16 ***
factor(day)76     -1.0674111  0.0201838 -52.884  < 2e-16 ***
factor(day)77     -1.0687169  0.0201812 -52.956  < 2e-16 ***
factor(day)78     -1.0700215  0.0201784 -53.028  < 2e-16 ***
factor(day)79     -1.0713876  0.0201752 -53.104  < 2e-16 ***
factor(day)80     -1.0728171  0.0201720 -53.183  < 2e-16 ***
factor(day)81     -1.0740684  0.0201693 -53.253  < 2e-16 ***
factor(day)82     -1.0753325  0.0201666 -53.322  < 2e-16 ***
factor(day)83     -1.0766590  0.0201639 -53.395  < 2e-16 ***
factor(day)84     -1.0776515  0.0201618 -53.450  < 2e-16 ***
factor(day)85     -1.0786049  0.0201599 -53.502  < 2e-16 ***
factor(day)86     -1.0795956  0.0201580 -53.557  < 2e-16 ***
factor(day)87     -1.0804462  0.0201563 -53.603  < 2e-16 ***
factor(day)88     -1.0811481  0.0201548 -53.642  < 2e-16 ***
factor(day)89     -1.0818946  0.0201533 -53.683  < 2e-16 ***
factor(day)90     -1.0826005  0.0201520 -53.722  < 2e-16 ***
factor(day)91     -1.0833290  0.0201505 -53.762  < 2e-16 ***
factor(day)92     -1.0841938  0.0201489 -53.809  < 2e-16 ***
factor(day)93     -1.0848968  0.0201475 -53.848  < 2e-16 ***
factor(day)94     -1.0858152  0.0201459 -53.898  < 2e-16 ***
factor(day)95     -1.0865534  0.0201444 -53.938  < 2e-16 ***
factor(day)96     -1.0872031  0.0201432 -53.974  < 2e-16 ***
factor(day)97     -1.0879391  0.0201419 -54.014  < 2e-16 ***
factor(day)98     -1.0885379  0.0201408 -54.046  < 2e-16 ***
factor(day)99     -1.0891227  0.0201397 -54.078  < 2e-16 ***
factor(day)100    -1.0896671  0.0201386 -54.108  < 2e-16 ***
---
Signif. codes:  0 ‘***’ 0.001 ‘**’ 0.01 ‘*’ 0.05 ‘.’ 0.1 ‘ ’ 1

(Dispersion parameter for Gamma family taken to be 0.59243)

    Null deviance: 240337  on 198465  degrees of freedom
Residual deviance: 126610  on 198360  degrees of freedom
AIC: 885146

Number of Fisher Scoring iterations: 6

> drop1(M2, test = "Chi")
Single term deletions

Model:
sqrtsld ~ factor(incr) + factor(depth) + factor(day)
               Df Deviance     AIC scaled dev.  Pr(>Chi)
<none>              126610  885146
factor(incr)    3   132570  895199       10059 < 2.2e-16 ***
```

```
factor(depth)   2   152138  928232       43090 < 2.2e-16 ***
factor(day)   100   199210 1007491      122545 < 2.2e-16 ***
---
Signif. codes:  0 ‘***’ 0.001 ‘**’ 0.01 ‘*’ 0.05 ‘.’ 0.1 ‘ ’ 1

> posth <- glht(M2, linfct=mcp(factor(incr) ="Tukey"), data = m2)
> summary(posth)

         Simultaneous Tests for General Linear Hypotheses

Multiple Comparisons of Means: Tukey Contrasts


Fit: glm(formula = sqrtsld ~ factor(incr)  + factor(depth)  + factor(day), family = Gam
    data = m2)

Linear Hypotheses:
                     Estimate Std. Error z value Pr(>|z|)
0.005 - 0.001 == 0 -0.0567097  0.0010646  -53.27   <2e-16 ***
0.01 - 0.001 == 0  -0.0761433  0.0010140  -75.09   <2e-16 ***
0.025 - 0.001 == 0 -0.0887582  0.0009907  -89.59   <2e-16 ***
0.01 - 0.005 == 0  -0.0194336  0.0008020  -24.23   <2e-16 ***
0.025 - 0.005 == 0 -0.0320485  0.0007702  -41.61   <2e-16 ***
0.025 - 0.01 == 0  -0.0126149  0.0006930  -18.20   <2e-16 ***
---
Signif. codes:  0 ‘***’ 0.001 ‘**’ 0.01 ‘*’ 0.05 ‘.’ 0.1 ‘ ’ 1
(Adjusted p values reported -- single-step method)

> posth2 <- glht(M2, linfct=mcp(factor(depth) ="Tukey"), data = m2)
> summary(posth2)

         Simultaneous Tests for General Linear Hypotheses

Multiple Comparisons of Means: Tukey Contrasts


Fit: glm(formula = sqrtsld ~ factor(incr)  + factor(depth)  + factor(day), family = Gam
    data = m2)

Linear Hypotheses:
                  Estimate Std. Error z value Pr(>|z|)
1000 - 700 == 0  0.0025041  0.0005788   4.326  3.1e-05 ***
1500 - 700 == 0  0.2393491  0.0014968 159.908  < 1e-05 ***
1500 - 1000 == 0 0.2368451  0.0014974 158.166  < 1e-05 ***
---
Signif. codes:  0 ‘***’ 0.001 ‘**’ 0.01 ‘*’ 0.05 ‘.’ 0.1 ‘ ’ 1
(Adjusted p values reported -- single-step method)
```

VERTICAL

(N.B. depth was excluded from this model due to collinearity with increment)

```
> summary(M2)

Call:
glm(formula = sqrtsld ~ factor(inc) + factor(day), family = Gamma, 
    data = m2)

Deviance Residuals: 
    Min       1Q   Median       3Q      Max  
-2.9643  -0.9474  -0.2938   0.3210   6.1526  

Coefficients:
                   Estimate Std. Error t value Pr(>|t|)    
(Intercept)        3.3872153  0.0745824  45.416   <2e-16 ***
factor(inc)201    -0.0907001  0.0025040 -36.222   <2e-16 ***
factor(inc)210    -0.1380758  0.0023017 -59.989   <2e-16 ***
factor(inc)250    -0.1580969  0.0022371 -70.670   <2e-16 ***
factor(inc)1000.1  0.1007400  0.0036654  27.484   <2e-16 ***
factor(inc)1001   -0.0338291  0.0028075 -12.049   <2e-16 ***
factor(inc)1010   -0.1150933  0.0023926 -48.104   <2e-16 ***
factor(inc)1050   -0.1475837  0.0022696 -65.027   <2e-16 ***
factor(inc)1750.1  3.0114618  0.0252754 119.146   <2e-16 ***
factor(inc)1751    1.0225954  0.0104667  97.700   <2e-16 ***
factor(inc)1760    0.2336372  0.0046002  50.788   <2e-16 ***
factor(inc)1800   -0.0001409  0.0030120  -0.047    0.963    
factor(day)2      -0.7770793  0.0941963  -8.250   <2e-16 ***
factor(day)3      -1.2282176  0.0884766 -13.882   <2e-16 ***
factor(day)4      -1.5217285  0.0851517 -17.871   <2e-16 ***
factor(day)5      -1.7153929  0.0831537 -20.629   <2e-16 ***
factor(day)6      -1.8840086  0.0815529 -23.102   <2e-16 ***
factor(day)7      -2.0203755  0.0803592 -25.142   <2e-16 ***
factor(day)8      -2.1407135  0.0793850 -26.966   <2e-16 ***
factor(day)9      -2.2497424  0.0785695 -28.634   <2e-16 ***
factor(day)10     -2.3247657  0.0780466 -29.787   <2e-16 ***
factor(day)11     -2.3937377  0.0775944 -30.849   <2e-16 ***
factor(day)12     -2.4557350  0.0772117 -31.805   <2e-16 ***
factor(day)13     -2.5131581  0.0768778 -32.690   <2e-16 ***
factor(day)14     -2.5610152  0.0766148 -33.427   <2e-16 ***
factor(day)15     -2.6027638  0.0763970 -34.069   <2e-16 ***
factor(day)16     -2.6349082  0.0762368 -34.562   <2e-16 ***
factor(day)17     -2.6722041  0.0760591 -35.133   <2e-16 ***
factor(day)18     -2.7080019  0.0758969 -35.680   <2e-16 ***
factor(day)19     -2.7346824  0.0757815 -36.086   <2e-16 ***
factor(day)20     -2.7561566  0.0756920 -36.413   <2e-16 ***
factor(day)21     -2.7807701  0.0755931 -36.786   <2e-16 ***
factor(day)22     -2.8040327  0.0755034 -37.138   <2e-16 ***
factor(day)23     -2.8285195  0.0754129 -37.507   <2e-16 ***
factor(day)24     -2.8481315  0.0753433 -37.802   <2e-16 ***
factor(day)25     -2.8679139  0.0752758 -38.099   <2e-16 ***
factor(day)26     -2.8869563  0.0752133 -38.384   <2e-16 ***
factor(day)27     -2.9010471  0.0751687 -38.594   <2e-16 ***
factor(day)28     -2.9142966  0.0751281 -38.791   <2e-16 ***
factor(day)29     -2.9278435  0.0750877 -38.992   <2e-16 ***
factor(day)30     -2.9416610  0.0750480 -39.197   <2e-16 ***
factor(day)31     -2.9566725  0.0750063 -39.419   <2e-16 ***
factor(day)32     -2.9711908  0.0749675 -39.633   <2e-16 ***
factor(day)33     -2.9833771  0.0749361 -39.812   <2e-16 ***
factor(day)34     -2.9933674  0.0749112 -39.959   <2e-16 ***
factor(day)35     -3.0032249  0.0748873 -40.103   <2e-16 ***
factor(day)36     -3.0132476  0.0748637 -40.250   <2e-16 ***
factor(day)37     -3.0224837  0.0748427 -40.384   <2e-16 ***
factor(day)38     -3.0306631  0.0748246 -40.504   <2e-16 ***
factor(day)39     -3.0380687  0.0748086 -40.611   <2e-16 ***
factor(day)40     -3.0446497  0.0747948 -40.707   <2e-16 ***
factor(day)41     -3.0520096  0.0747797 -40.813   <2e-16 ***
```

```
factor(day)42     -3.0588860  0.0747660 -40.913   <2e-16 ***
factor(day)43     -3.0654184  0.0747533 -41.007   <2e-16 ***
factor(day)44     -3.0714058  0.0747419 -41.093   <2e-16 ***
factor(day)45     -3.0765639  0.0747324 -41.168   <2e-16 ***
factor(day)46     -3.0812286  0.0747239 -41.235   <2e-16 ***
factor(day)47     -3.0855265  0.0747162 -41.297   <2e-16 ***
factor(day)48     -3.0891542  0.0747099 -41.349   <2e-16 ***
factor(day)49     -3.0932443  0.0747029 -41.407   <2e-16 ***
factor(day)50     -3.0971918  0.0746962 -41.464   <2e-16 ***
factor(day)51     -3.1011320  0.0746897 -41.520   <2e-16 ***
factor(day)52     -3.1045822  0.0746841 -41.570   <2e-16 ***
factor(day)53     -3.1086838  0.0746776 -41.628   <2e-16 ***
factor(day)54     -3.1128548  0.0746711 -41.688   <2e-16 ***
factor(day)55     -3.1167956  0.0746651 -41.744   <2e-16 ***
factor(day)56     -3.1202542  0.0746599 -41.793   <2e-16 ***
factor(day)57     -3.1232950  0.0746555 -41.836   <2e-16 ***
factor(day)58     -3.1261116  0.0746514 -41.876   <2e-16 ***
factor(day)59     -3.1289025  0.0746474 -41.916   <2e-16 ***
factor(day)60     -3.1311733  0.0746443 -41.948   <2e-16 ***
factor(day)61     -3.1333393  0.0746413 -41.979   <2e-16 ***
factor(day)62     -3.1355828  0.0746382 -42.010   <2e-16 ***
factor(day)63     -3.1377281  0.0746354 -42.041   <2e-16 ***
factor(day)64     -3.1399558  0.0746324 -42.072   <2e-16 ***
factor(day)65     -3.1422715  0.0746294 -42.105   <2e-16 ***
factor(day)66     -3.1445478  0.0746265 -42.137   <2e-16 ***
factor(day)67     -3.1467759  0.0746237 -42.169   <2e-16 ***
factor(day)68     -3.1489032  0.0746211 -42.199   <2e-16 ***
factor(day)69     -3.1507857  0.0746188 -42.225   <2e-16 ***
factor(day)70     -3.1525528  0.0746166 -42.250   <2e-16 ***
factor(day)71     -3.1542676  0.0746146 -42.274   <2e-16 ***
factor(day)72     -3.1558570  0.0746127 -42.296   <2e-16 ***
factor(day)73     -3.1575707  0.0746108 -42.321   <2e-16 ***
factor(day)74     -3.1592156  0.0746089 -42.344   <2e-16 ***
factor(day)75     -3.1607378  0.0746072 -42.365   <2e-16 ***
factor(day)76     -3.1620772  0.0746057 -42.384   <2e-16 ***
factor(day)77     -3.1633698  0.0746042 -42.402   <2e-16 ***
factor(day)78     -3.1644426  0.0746031 -42.417   <2e-16 ***
factor(day)79     -3.1655429  0.0746019 -42.432   <2e-16 ***
factor(day)80     -3.1667674  0.0746006 -42.450   <2e-16 ***
factor(day)81     -3.1679064  0.0745994 -42.466   <2e-16 ***
factor(day)82     -3.1691018  0.0745981 -42.482   <2e-16 ***
factor(day)83     -3.1702304  0.0745970 -42.498   <2e-16 ***
factor(day)84     -3.1713557  0.0745958 -42.514   <2e-16 ***
factor(day)85     -3.1721976  0.0745950 -42.526   <2e-16 ***
factor(day)86     -3.1731809  0.0745940 -42.539   <2e-16 ***
factor(day)87     -3.1740587  0.0745931 -42.552   <2e-16 ***
factor(day)88     -3.1749601  0.0745922 -42.564   <2e-16 ***
factor(day)89     -3.1758415  0.0745914 -42.577   <2e-16 ***
factor(day)90     -3.1767060  0.0745905 -42.589   <2e-16 ***
factor(day)91     -3.1775425  0.0745897 -42.600   <2e-16 ***
factor(day)92     -3.1782262  0.0745891 -42.610   <2e-16 ***
factor(day)93     -3.1789290  0.0745884 -42.620   <2e-16 ***
factor(day)94     -3.1797641  0.0745877 -42.631   <2e-16 ***
factor(day)95     -3.1803831  0.0745871 -42.640   <2e-16 ***
factor(day)96     -3.1808299  0.0745867 -42.646   <2e-16 ***
factor(day)97     -3.1816287  0.0745859 -42.657   <2e-16 ***
factor(day)98     -3.1820690  0.0745855 -42.663   <2e-16 ***
factor(day)99     -3.1827886  0.0745849 -42.673   <2e-16 ***
factor(day)100    -3.1834465  0.0745842 -42.683   <2e-16 ***
---
Signif. codes:  0 ‘***’ 0.001 ‘**’ 0.01 ‘*’ 0.05 ‘.’ 0.1 ‘ ’ 1

(Dispersion parameter for Gamma family taken to be 1.032367)

    Null deviance: 473844  on 230102  degrees of freedom
Residual deviance: 223173  on 229992  degrees of freedom
AIC: 861369

Number of Fisher Scoring iterations: 7
```

```
> drop1(M2, test = "Chi")

Model:
sqrtsld ~ factor(inc) + factor(day)
            Df Deviance    AIC scaled dev.  Pr(>Chi)
<none>           223173 861369
factor(inc) 11   355700 989719      128372 < 2.2e-16 ***
factor(day) 99   316287 951366       90195 < 2.2e-16 ***
---
Signif. codes:  0 ‘***’ 0.001 ‘**’ 0.01 ‘*’ 0.05 ‘.’ 0.1 ‘ ’ 1

> posth <- glht(M2, linfct=mcp(factor(inc)="Tukey"), data = m2)
> summary(posth)

       Simultaneous Tests for General Linear Hypotheses

Multiple Comparisons of Means: Tukey Contrasts


Fit: glm(formula = sqrtsld ~ factor(inc) + factor(day), family = Gamma,
    data = m2)

Linear Hypotheses:
                       Estimate Std. Error  z value Pr(>|z|)
201 - 200.1 == 0     -0.0907001  0.0025040  -36.222   <1e-10 ***
210 - 200.1 == 0     -0.1380758  0.0023017  -59.989   <1e-10 ***
250 - 200.1 == 0     -0.1580969  0.0022371  -70.670   <1e-10 ***
1000.1 - 200.1 == 0   0.1007400  0.0036654   27.484   <1e-10 ***
1001 - 200.1 == 0    -0.0338291  0.0028075  -12.049   <1e-10 ***
1010 - 200.1 == 0    -0.1150933  0.0023926  -48.104   <1e-10 ***
1050 - 200.1 == 0    -0.1475837  0.0022696  -65.027   <1e-10 ***
1750.1 - 200.1 == 0   3.0114618  0.0252754  119.146   <1e-10 ***
1751 - 200.1 == 0     1.0225954  0.0104667   97.700   <1e-10 ***
1760 - 200.1 == 0     0.2336372  0.0046002   50.788   <1e-10 ***
1800 - 200.1 == 0    -0.0001409  0.0030120   -0.047        1
210 - 201 == 0       -0.0473757  0.0015754  -30.072   <1e-10 ***
250 - 201 == 0       -0.0673968  0.0014769  -45.634   <1e-10 ***
1000.1 - 201 == 0     0.1914401  0.0032656   58.623   <1e-10 ***
1001 - 201 == 0       0.0568710  0.0022566   25.202   <1e-10 ***
1010 - 201 == 0      -0.0243933  0.0017077  -14.285   <1e-10 ***
1050 - 201 == 0      -0.0568837  0.0015270  -37.252   <1e-10 ***
1750.1 - 201 == 0     3.1021619  0.0252220  122.994   <1e-10 ***
1751 - 201 == 0       1.1132955  0.0103358  107.712   <1e-10 ***
1760 - 201 == 0       0.3243373  0.0042900   75.603   <1e-10 ***
1800 - 201 == 0       0.0905592  0.0025076   36.114   <1e-10 ***
250 - 210 == 0       -0.0200211  0.0010840  -18.470   <1e-10 ***
1000.1 - 210 == 0     0.2388158  0.0031147   76.674   <1e-10 ***
1001 - 210 == 0       0.1042467  0.0020287   51.386   <1e-10 ***
1010 - 210 == 0       0.0229824  0.0013872   16.568   <1e-10 ***
1050 - 210 == 0      -0.0095080  0.0011532   -8.245   <1e-10 ***
1750.1 - 210 == 0     3.1495376  0.0252037  124.963   <1e-10 ***
1751 - 210 == 0       1.1606712  0.0102904  112.791   <1e-10 ***
1760 - 210 == 0       0.3717130  0.0041772   88.987   <1e-10 ***
1800 - 210 == 0       0.1379349  0.0023056   59.827   <1e-10 ***
1000.1 - 250 == 0     0.2588369  0.0030680   84.366   <1e-10 ***
1001 - 250 == 0       0.1242678  0.0019545   63.579   <1e-10 ***
1010 - 250 == 0       0.0430036  0.0012728   33.785   <1e-10 ***
1050 - 250 == 0       0.0105131  0.0010095   10.415   <1e-10 ***
1750.1 - 250 == 0     3.1695587  0.0251983  125.785   <1e-10 ***
1751 - 250 == 0       1.1806923  0.0102770  114.887   <1e-10 ***
1760 - 250 == 0       0.3917341  0.0041430   94.554   <1e-10 ***
1800 - 250 == 0       0.1579560  0.0022411   70.481   <1e-10 ***
1001 - 1000.1 == 0   -0.1345691  0.0035025  -38.421   <1e-10 ***
1010 - 1000.1 == 0   -0.2158333  0.0031817  -67.836   <1e-10 ***
1050 - 1000.1 == 0   -0.2483237  0.0030914  -80.328   <1e-10 ***
1750.1 - 1000.1 == 0  2.9107218  0.0253603  114.775   <1e-10 ***
1751 - 1000.1 == 0    0.9218555  0.0106713   86.386   <1e-10 ***
1760 - 1000.1 == 0    0.1328972  0.0050518   26.307   <1e-10 ***
```

```
1800 - 1000.1 == 0   -0.1008808  0.0036679  -27.504   <1e-10 ***
1010 - 1001 == 0     -0.0812642  0.0021318  -38.120   <1e-10 ***
1050 - 1001 == 0     -0.1137547  0.0019919  -57.108   <1e-10 ***
1750.1 - 1001 == 0    3.0452909  0.0252529  120.592   <1e-10 ***
1751 - 1001 == 0      1.0564245  0.0104116  101.466   <1e-10 ***
1760 - 1001 == 0      0.2674663  0.0044720   59.809   <1e-10 ***
1800 - 1001 == 0      0.0336882  0.0028107   11.986   <1e-10 ***
1050 - 1010 == 0     -0.0324904  0.0013314  -24.404   <1e-10 ***
1750.1 - 1010 == 0    3.1265552  0.0252117  124.012   <1e-10 ***
1751 - 1010 == 0      1.1376888  0.0103103  110.345   <1e-10 ***
1760 - 1010 == 0      0.3487305  0.0042269   82.502   <1e-10 ***
1800 - 1010 == 0      0.1149525  0.0023964   47.970   <1e-10 ***
1750.1 - 1050 == 0    3.1590456  0.0252010  125.354   <1e-10 ***
1751 - 1050 == 0      1.1701792  0.0102837  113.790   <1e-10 ***
1760 - 1050 == 0      0.3812210  0.0041600   91.639   <1e-10 ***
1800 - 1050 == 0      0.1474429  0.0022735   64.852   <1e-10 ***
1751 - 1750.1 == 0   -1.9888664  0.0271801  -73.174   <1e-10 ***
1760 - 1750.1 == 0   -2.7778246  0.0255102 -108.891   <1e-10 ***
1800 - 1750.1 == 0   -3.0116027  0.0252758 -119.150   <1e-10 ***
1760 - 1751 == 0     -0.7889582  0.0110246  -71.564   <1e-10 ***
1800 - 1751 == 0     -1.0227363  0.0104676  -97.705   <1e-10 ***
1800 - 1760 == 0     -0.2337781  0.0046022  -50.797   <1e-10 ***
---
Signif. codes:  0 ‘***’ 0.001 ‘**’ 0.01 ‘*’ 0.05 ‘.’ 0.1 ‘ ’ 1
(Adjusted p values reported -- single-step method)
```
